# Comparison of noninvasive cardiac output and stroke volume measurements using electrical impedance tomography with invasive methods in a swine model

**DOI:** 10.1038/s41598-024-53488-0

**Published:** 2024-02-05

**Authors:** Chi Ryang Chung, Ryoung Eun Ko, Geuk Young Jang, Kyounghun Lee, Gee Young Suh, Yongmin Kim, Eung Je Woo

**Affiliations:** 1 Department of Critical Care Medicine, Samsung Medical Center, Sungkyunkwan University School of Medicine, Seoul, Korea; 2 Department of Biomedical Engineering, College of Medicine, Kyung Hee University, 26 Kyungheedae-ro, Dongdaemun-gu, Seoul, 02447 Korea; 3 Department of Convergence IT Engineering, POSTECH, Pohang, Korea

**Keywords:** Preclinical research, Translational research

## Abstract

Pulmonary artery catheterization (PAC) has been used as a clinical standard for cardiac output (CO) measurements on humans. On animals, however, an ultrasonic flow sensor (UFS) placed around the ascending aorta or pulmonary artery can measure CO and stroke volume (SV) more accurately. The objective of this paper is to compare CO and SV measurements using a noninvasive electrical impedance tomography (EIT) device and three invasive devices using UFS, PAC-CCO (continuous CO) and arterial pressure-based CO (APCO). Thirty-two pigs were anesthetized and mechanically ventilated. A UFS was placed around the pulmonary artery through thoracotomy in 11 of them, while the EIT, PAC-CCO and APCO devices were used on all of them. Afterload and contractility were changed pharmacologically, while preload was changed through bleeding and injection of fluid or blood. Twenty-three pigs completed the experiment. Among 23, the UFS was used on 7 pigs around the pulmonary artery. The percentage error (PE) between CO_UFS_ and CO_EIT_ was 26.1%, and the 10-min concordance was 92.5%. Between SV_UFS_ and SV_EIT_, the PE was 24.8%, and the 10-min concordance was 94.2%. On analyzing the data from all 23 pigs, the PE between time-delay-adjusted CO_PAC-CCO_ and CO_EIT_ was 34.6%, and the 10-min concordance was 81.1%. Our results suggest that the performance of the EIT device in measuring dynamic changes of CO and SV on mechanically-ventilated pigs under different cardiac preload, afterload and contractility conditions is at least comparable to that of the PAC-CCO device. Clinical studies are needed to evaluate the utility of the EIT device as a noninvasive hemodynamic monitoring tool.

## Introduction

Cardiac output (CO) is a key parameter for hemodynamic assessment and monitoring in the critically-ill patients^1–3^. The thermodilution with pulmonary artery catheterization (PAC) using a Swan-Ganz catheter is currently considered as the clinical standard^4,5^. The PAC-ICO (intermittent CO) method requires a bolus injection of cold saline as a thermal indicator to measure CO intermittently, and it takes tens of seconds to get one CO data. The PAC-CCO (continuous CO) uses a heating coil placed on the catheter to repeatedly produce heat and measure a CO value every 60 s or slower. Both PAC methods are used to measure CO from selected patients, e.g., in operating room (OR) and cardiac catheterization laboratory^6^. Less invasive, but less accurate, methods using transpulmonary thermodilution (TPTD)^7^, pulse contour analysis (PCA)^8^ and arterial pressure-based cardiac output (APCO)^9^ are frequently used for patients in OR and intensive care unit (ICU). Both PCA and APCO methods provide a stroke volume (SV) measurement as often as every 20 s, from which CO is derived. The TPTD, PCA and APCO methods, although less invasive than PAC, still require catheters placed in both central vein and artery or in artery only. The currently-available gold standard in CO and SV measurements is an ultrasonic flow sensor (UFS) placed around the ascending aorta or pulmonary artery although its use is mostly limited to animals^10,11^.

Recently, PCA algorithms were combined with noninvasive methods to continuously measure arterial pressure using a finger cuff to estimate SV in every 20 s^12–14^. Although multiple devices based on these noninvasive methods have been used on patients without an arterial line, their accuracy in SV measurements would be potentially lower than that of an invasive PCA device since central blood pressure needs to be estimated from the peripheral blood pressure signals measured by a finger cuff. In addition, the duration of its use could be limited to a few hours or tens of minutes since some patients may not endure repeated inflation and deflation of a finger cuff for an extended period of time. Ultrasound echocardiography can be used to measure the left ventricular outflow tract velocity time integral as a surrogate for SV. However, the intra-operator and inter-operator variability and inability to use continuously are its disadvantages.

As a noninvasive method, impedance cardiography was first introduced in the 1970s^15–17^, but its clinical acceptance over the last 50 years has been slow mainly due to limited accuracy, especially on hemodynamically-unstable patients^18,19^. Instead of single or dual channels used in impedance cardiography, electrical impedance tomography (EIT) acquires more than 100 channels of impedance data with 16 to 32 electrodes placed around the chest^20–22^. Utilizing this large amount of measured data, EIT can produce cross-sectional images of an electrical conductivity distribution inside a thorax. In the last decade, it has seen its first clinical use as a tool to assess regional lung ventilation in mechanically-ventilated patients^23^.

Although several EIT-based methods have been attempted to quantify hemodynamic parameters^24–26^, cardiac EIT is not yet clinically available. One of the main difficulties was in extracting cardiogenic components embedded in measured multi-channel impedance data. In previous research to measure SV or CO using EIT, cardiac signal components were extracted from either a heart region-of-interest (ROI)^27^ or lung ROI^28^ in reconstructed EIT images. In a clinical study focusing on dynamic changes in stroke volume due to fluid administration, cardiac signal components were extracted reasonably well from lung ROIs in ECG-gated EIT images^29^.

Given the limited spatial resolution of EIT, it could be advantageous to extract a cardiac signal component directly from measured multi-channel impedance data rather than reconstructed EIT images. Recently, successful extraction of weak cardiogenic components (less than 10% of ventilatory components) in measured multi-channel impedance data was realized^30–32^ using novel algorithms to separate air and blood components. To assess the feasibility of a novel EIT device providing hemodynamic monitoring functions in addition to ventilation imaging functions, this EIT-based hemodynamic monitoring method needs to be evaluated in terms of its performance in CO and SV measurements through animal and also clinical studies.

In this paper, we performed two animal studies using a newly-developed EIT-based hemodynamic monitor. The objectives of this study were to (1) simultaneously measure CO and SV using EIT, UFS, PAC-CCO and APCO devices on pigs under different preload, afterload and contractility conditions and (2) compare the performance of the EIT device in measuring CO and SV with that of the invasive devices.

## Methods

We conducted two animal studies. The first one was performed at the Laboratory Animal Research Center of the Samsung Medical Center (SMC, Seoul, Korea) from March 19, 2021 to June 9, 2021. This study was reviewed and approved by the Institutional Animal Care and Use Committee (IACUC) of the Samsung Biomedical Research Institute (SBRI) (SMC-20210120003). The second animal study was conducted at the Biomedical Research Institute of the Seoul National University Bundang Hospital (SNUBH, Seongnam, Korea) from December 10, 2021 to March 3, 2022. This second study was reviewed and approved by the IACUC of the SBRI (SMC-20210923001) and the Biomedical Research Institute of the SNUBH (BA-2112-333-005-01). A total of 32 pigs (weights of 60–80 kg, at least 5 months old) were used in two animal studies (21 and 11 in the first and second study, respectively). There were no human participants involved in the study. All experiments were performed in accordance with relevant guidelines and regulations. The study is reported in accordance with ARRIVE guidelines (https://arriveguidelines.org/).

The goal of the first study was to evaluate the performance of the EIT-based hemodynamic monitor in comparison with PAC-CCO and APCO, which are most-widely used in ICU and OR. After the first study, the performance of the EIT-based hemodynamic monitor was further assessed in comparison with UFS around the pulmonary artery, which is considered to be most accurate. Both PAC-CCO and APCO were used again in the second study to effectively increase the sample size of the first study. The PAC-ICO method was not used in our study since (1) it was not available to us at the time of the animal studies and (2) it is rarely used clinically in ICU and OR lately. Furthermore, in our study using animals, UFS placed around the pulmonary artery via thoracotomy would be the best gold standard method since it is more accurate than PAC-ICO. In addition, UFS provides continuously-measured CO and SV data that can be directly compared with simultaneously-measured CO and SV data using the EIT-based hemodynamic monitor.

### Animal preparations

In both studies, the animal was premedicated with intramuscular injection of ketamine (20 mg/kg) and xylazine (2.5 mg/kg), and connected to an anesthesia machine (Fabius GS Premium, Drager, Germany) by tracheal intubation. Anesthesia was maintained by 2% isoflurane throughout the experiment. A Swan-Ganz catheter (774F75, Edwards Lifesciences, U.S.) was inserted in the pulmonary artery. In addition, a catheter (VLVFC416, Edwards Lifesciences, U.S.) was inserted in the right femoral artery, and another catheter (X3820ST, Edwards Lifesciences, U.S.) was inserted in the central vein. In the second study on 11 pigs, a UFS (T402-PB, Transonic, U.S.) was placed around the pulmonary artery through a thoracotomy, followed by chest closure. Hairs were removed, and the pig’s skin was scrubbed with an abrasive gel around the fifth intercostal space before attaching 16 electrodes (2560 Red Dot, 3M, U.S.) on the chest for EIT data acquisition. For ECG measurements, additional electrodes (2560 Red Dot, 3M, U.S.) were attached to the right and left legs.

### Interventions

**Figure 1:**
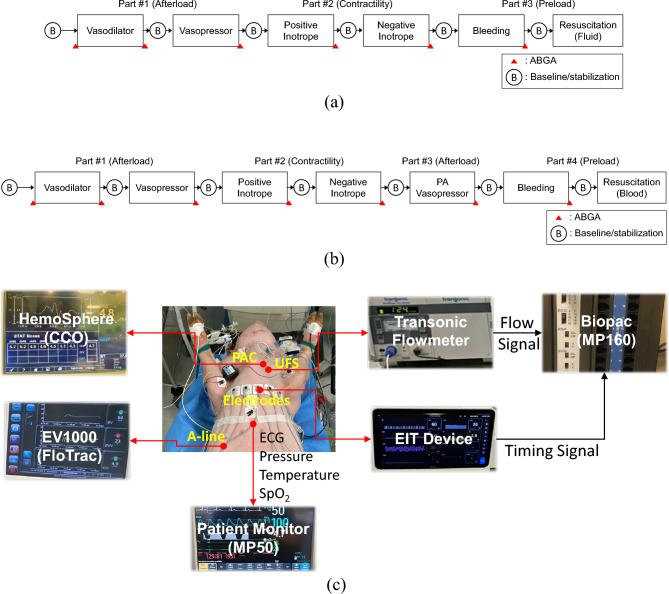
Interventions and sequence used in the (**a**) first and (**b**) second animal study. Details are described in the online supplementary materials (Sections S1 and S2). (**c**) Experimental setup. The ultrasonic flow sensor (UFS) connected to the Transonic flowmeter was placed around the pulmonary artery in the second study.

Figure 1a,b show an intervention sequence to effect changes in preload, afterload and contractility during an experiment in the first and second animal study, respectively. The mean arterial pressure (MAP) was maintained at 70–80 mmHg for at least 10 min at the beginning of the experiment. An arterial blood gas analysis (ABGA) was performed before each intervention during the experiment. The pH value was kept between 7.4 and 7.5 by adjusting the tidal volume and/or respiration rate of the anesthesia machine. After each study, the animal was euthanized by intravenous injection of potassium chloride. More details about the interventions in both studies are described in the online supplementary materials (Sections S1 and S2).

### Measurements

An investigational EIT device (HemoVista, BiLab, Korea) with 16 electrodes was used for CO_EIT_ and SV_EIT_ measurements as described in the online supplementary materials (Section S4). In both animal studies, the first reference device was either the Vigilance II-CCO (Edwards Lifesciences, U.S.) or HemoSphere-CCO (Edwards Lifesciences, U.S.) using the PAC-CCO method with a Swan-Ganz catheter to measure CO_PAC-CCO_ and SV_PAC-CCO_. The second reference device was the EV1000-FloTrac (Edwards Lifesciences, U.S.) using the APCO method with an arterial catheter to measure CO_APCO_ and SV_APCO_. In the second animal study with 11 animals, the third reference device was the UFS (T402-PB, Transonic, U.S.) placed around the pulmonary artery. Since all measurements were acquired simultaneously and independently on each animal using separate devices, no control group was used. For the same reason, neither randomization nor blinding in animal subject selection was necessary.

ECG, SpO_2_, body temperature and 3 channels of invasive blood pressure signals from catheters in the pulmonary artery, femoral artery and central vein were recorded using the patient monitor (MP50, Philips Healthcare, The Netherlands). The MP160 (Biopac, U.S.) was used not only for digitizing the analog output signal from the UFS, but also for data synchronization between the UFS and EIT devices. Figure 1c shows these various devices used in the experiment.

### Data extraction and synchronization

The measured CO_EIT_ and SV_EIT_ values were displayed in real time and stored in the EIT device every 5 s. The stored data were extracted as a data file through the USB port of the EIT device at the end of each experiment. The data from the HemoSphere-CCO or Vigilance II-CCO and MP50 were transferred in real time to a laptop computer running VitalRecorder (VitalDB, https://vitaldb.net/) and stored there. The time-stamped data from the EV1000-FloTrac were extracted as a data file through the USB port of the EV1000-FloTrac at the end of each experiment. The flow data extracted from the UFS device after each experiment were used to compute an SV_UFS_ value for each cardiac cycle, and then an average value during the most-recent 5 s interval was obtained for subsequent data analyses.

For proper data analysis, it is important to align the extracted data from the multiple devices in time. Since the EIT device was synchronized with the MP160 via a timing signal, SV_EIT_/CO_EIT_ data could be time-aligned with SV_UFS_/CO_UFS_ using the timing signal. The data from the MP50 were aligned with the data from the MP160 by maximizing the cross-correlation in HR over the entire study period for each experiment. Similarly, the data from the EV1000-FloTrac were aligned with the data from the MP160 by maximizing the cross-correlation in HR over the entire study period for each experiment.

The CO_PAC-CCO_ data have a varying internal time delay since the PAC-CCO device uses a thermodilution method with data averaging and processing. Since this time-delay information was unavailable, it was estimated for each animal in the first study by minimizing the relative standard deviation of the differences between the MAP data and the time-delayed CO_PAC-CCO_ data. In the second study, the CO_UFS_ data were used for estimating a time delay instead of the MAP data.

### Reference device and volume calibration

The PAC-CCO and UFS device was chosen as a reference device (REF) in the first and second study, respectively, to assess the performance of each device under test (DUT), i.e., EIT and APCO in the first study and EIT, PAC-CCO and APCO in the second study. The EIT device used in these animal studies measured relative SV_EIT_ values in an arbitrary unit rather than absolute SV_EIT_ values in the unit of mL, although its clinical version is now equipped with an SV formula to output absolute SV_EIT_ values using patient’s demographic and other information. Therefore, relative SV_EIT_ values measured from the animals were scaled to absolute values using simultaneously-measured SV data from a reference device.

In the first animal study, we determined a scale factor to convert the relative SV_EIT_ values to absolute values using the SV_PAC-CCO_ data. This minimized the bias in the absolute SV_EIT_ values with respect to the SV_PAC-CCO_ data. Since there was a different amount of bias between the PAC-CCO and APCO data, we minimized this bias by scaling the SV_APCO_ data for each animal using the same SV_PAC-CCO_ data used to scale the SV_EIT_ data. In the second study, we determined a scale factor to convert the relative SV_EIT_ values to absolute SV_EIT_ values using the SV_UFS_ data. Again, there were different amounts of bias among the UFS, PAC-CCO and APCO data. We, therefore, used the same SV_UFS_ data used to scale the SV_EIT_ data to similarly scale each of the SV_PAC-CCO_ and SV_APCO_ data from each animal.

To compute such a scale factor, a pair of measured data with REF (PAC-CCO or UFS) and DUT (EIT, APCO or PAC-CCO) during the baseline/stabilization part at the beginning of each experiment was used. Each scale factor was computed as ‘ScaleFactor = SV_REF_(t_0_)/SV_DUT_(t_0_)’ where t_0_ is the time of the first data pair (SV_REF_, SV_DUT_). For the remaining SV_DUT_ data, SV_DUT,Scaled_ was computed as ScaleFactor × SV_DUT_. No offset value was used in this volume calibration.

### Statistical analyses

We used the NCSS 2021 software (NCSS Statistical Software, U.S.) for the Bland–Altman analysis^33^ and the Matlab software (MathWorks, U.S.) for the concordance analysis^34^. For both Bland–Altman and concordance analyses, 12 pairs of measured data $\left({{\text{X}}}_{{\text{REF}}},{{\text{X}}}_{{\text{DUT}}}\right)$ were used where ${\text{X}}$ was either CO or SV: $\left({{\text{CO}}}_{{\text{UFS}}},{{\text{CO}}}_{{\text{EIT}}}\right)$, $\left({{\text{CO}}}_{{\text{UFS}}},{{\text{CO}}}_{{\text{PAC}}-{\text{CCO}}}\right)$, $\left({{\text{CO}}}_{{\text{UFS}}},{{\text{CO}}}_{{\text{APCO}}}\right)$, $\left({{\text{SV}}}_{{\text{UFS}}},{{\text{SV}}}_{{\text{EIT}}}\right)$, $\left({{\text{SV}}}_{{\text{UFS}}},{{\text{SV}}}_{{\text{PAC}}-{\text{CCO}}}\right)$, $\left({{\text{SV}}}_{{\text{UFS}}},{{\text{SV}}}_{{\text{APCO}}}\right)$, $\left({{\text{CO}}}_{{\text{PAC}}-{\text{CCO}}},{{\text{CO}}}_{{\text{EIT}}}\right)$, $\left({{\text{CO}}}_{{\text{PAC}}-{\text{CCO}}},{{\text{CO}}}_{{\text{APCO}}}\right)$, $\left({{\text{CO}}}_{{\text{APCO}}},{{\text{CO}}}_{{\text{EIT}}}\right)$, $\left({{\text{SV}}}_{{\text{PAC}}-{\text{CCO}}},{{\text{SV}}}_{{\text{EIT}}}\right)$, $\left({{\text{SV}}}_{{\text{PAC}}-{\text{CCO}}},{{\text{SV}}}_{{\text{APCO}}}\right)$ and $\left({{\text{SV}}}_{{\text{APCO}}},{{\text{SV}}}_{{\text{EIT}}}\right)$. In the Bland–Altman analysis, different numbers of data points per animal were incorporated as weights, and we computed the bias and 95% limits of agreement (95% LoA) with their 95% confidence intervals (95% CI). The percentage error (PE) was computed using the method described in the online supplementary materials (Section S5). The condition of concordance and its computation are also described in the online supplementary materials (Section S5).

### Acceptance criteria for interchangeability

Performance evaluation and comparison of different hemodynamic monitors have been investigated in meta-analyses^35,36^, reviews^37–39^ and method standardization studies^40–43^. In most studies, PAC-ICO was chosen as the clinical gold standard for CO measurements. Assuming that the error of the PAC-ICO method itself is about 20%, the PE of 30% (about $\sqrt{2}\times 20$%) was suggested as an acceptance criterion for interchangeability of a new device with PAC-ICO^35^. In this study, we used UFS rather than PCA-ICO. However, the interchangeability of EIT with PAC-ICO could be assessed through the PE between ${{\text{CO}}}_{{\text{EIT}}}$ and ${{\text{CO}}}_{{\text{UFS}}}$. Since the error of the UFS method itself is smaller than or equal to that of PAC-ICO (20%), the PE of 30% between ${{\text{CO}}}_{{\text{EIT}}}$ and ${{\text{CO}}}_{{\text{UFS}}}$ can be used as the acceptance criteria for interchangeability between EIT and PAC-ICO. With an estimated error of around 30% associated with PAC-CCO^44–46^, a different acceptance criterion of 42% (about $\sqrt{2}\times 30$%) could be established for interchangeability with PAC-CCO. It should be noted that the 20% error of the PAC-ICO method could be achieved only in a highly-controlled environment^47,48^. Since the error of the PAC-ICO method in real clinical environments is higher than 20%, the PE of 42% could be considered as a practical acceptance criterion for clinical equivalence of different CO devices in practice.

### Ethical approval

The first animal study was approved by the IACUC of the Samsung Biomedical Research Institute (SBRI) (SMC-20210120003). The second animal study was approved by the IACUC of the SBRI (SMC-20210923001) and the Biomedical Research Institute of the SNUBH (BA-2112-333-005-01).

## Results

The information about all 32 animal experiments can be found in the online supplementary materials (Table S1 in Section S6). Pigs #1–#21 were used in the first study, while pigs #22–#32 were used in the second study. The data from 9 pigs, #1–#4, #10, #22, #24, #29 and #32, were excluded from data analyses. Pigs #1, #2, #4 and #22 were used as pilots to find proper ranges of dosage for vasodilator, vasopressor, positive inotrope and negative inotrope. Animal preparations failed in the pig #3. In case of the pig #10, the EIT data were not properly captured. The thoracotomy procedure on pigs #24 and #29 failed. In case of the pig #32, the UFS data were not properly captured. Although the Vigilance II-CCO was used on pigs #5 and #6 and the HemoSphere-CCO was used on pigs #7–#32, pigs #5 and #6 were included in data analyses since these two devices from the same manufacturer are considered to be equivalent in measuring CO values.

The dosages of vasodilator, vasopressor, positive inotrope and negative inotrope varied greatly among animals as shown in Table S1 in the online supplementary materials (Sect. S6). Although the amount of fluid provided during volume resuscitation was 1 L for all 21 pigs in the first animal study, the amount of bleeding was quite different for each animal. In the second animal study, the amount of blood provided during volume resuscitation was the same as the amount of removed blood, which was also quite different for each animal. Since these interventions were determined by MAP, the inter-animal hemodynamic variability was large.

**Figure 2:**
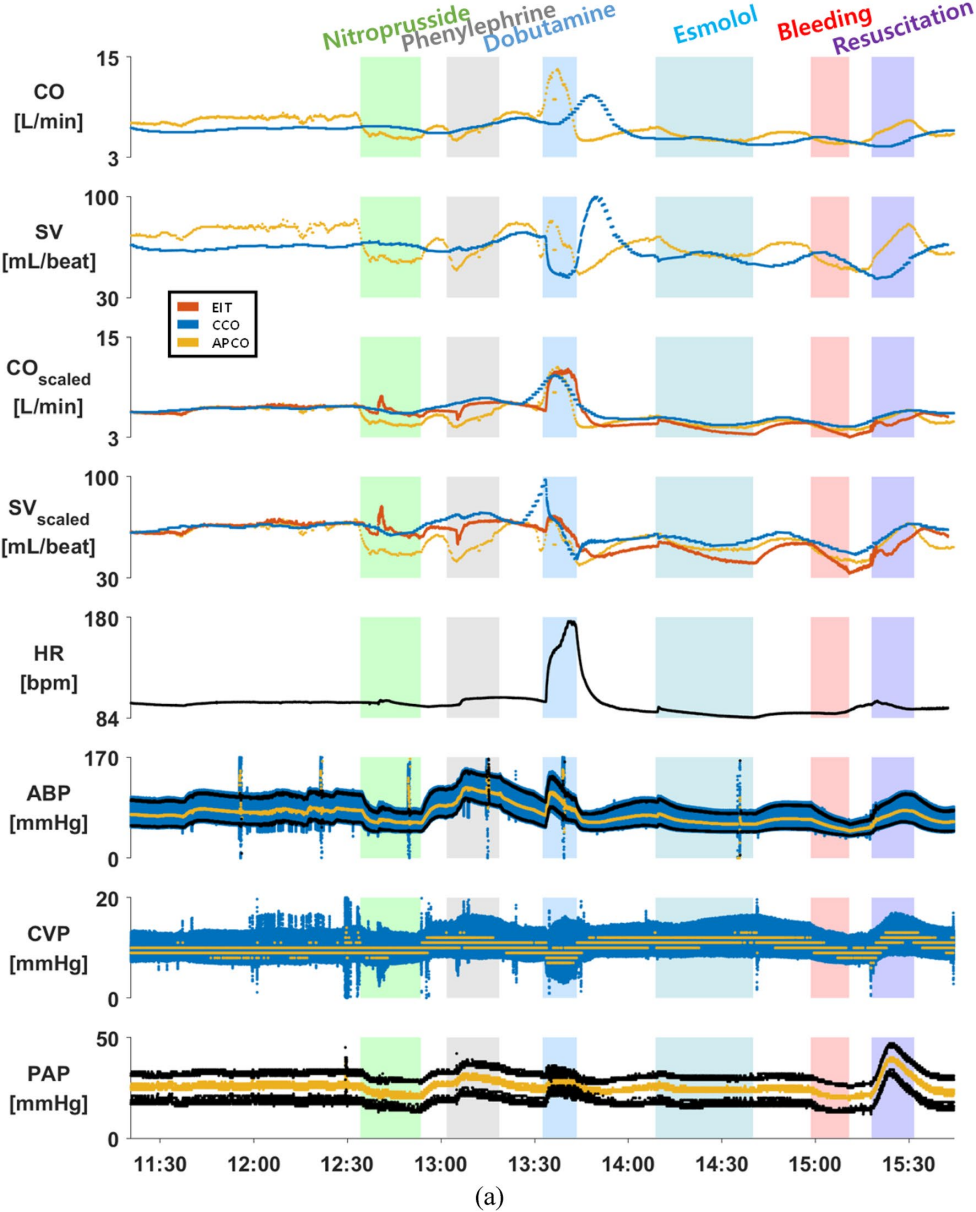

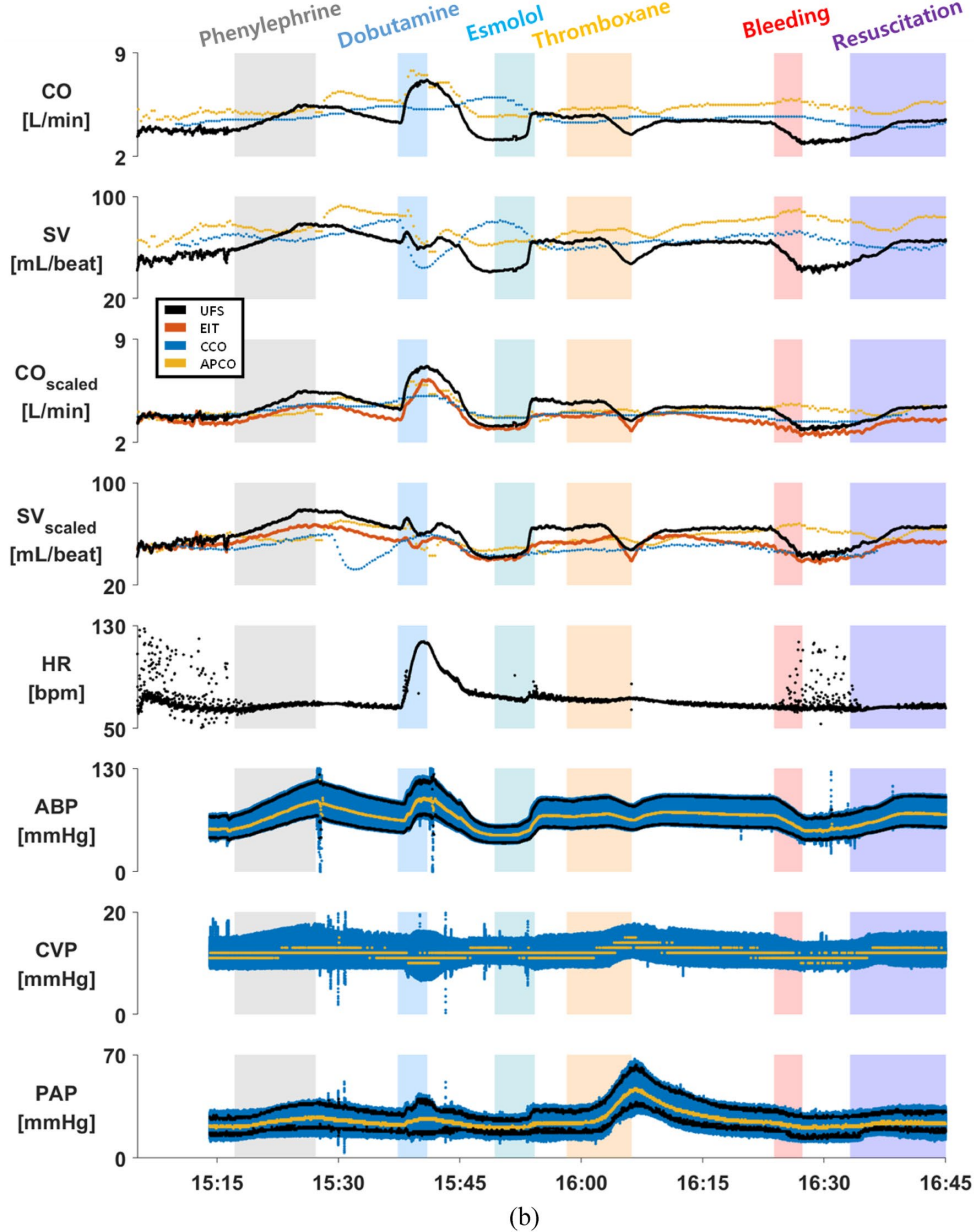
(**a**) An example of the measured CO, SV, CO_Scaled_, SV_Scaled_, HR, ABP, CVP and PAP data from the pig #9 in the first animal study. In the CO_Scaled_ and SV_Scaled_ plots, the EIT and APCO data were scaled using a PAC-CCO datum in the beginning of each experiment as a reference value after a time-delay adjustment was applied to the PAC-CCO data. (**b**) An example of the measured CO, SV, CO_Scaled_, SV_Scaled_, HR, ABP, CVP and PAP data from the pig #27 in the second animal study. In the CO_Scaled_ and SV_Scaled_ plots, the EIT, PAC-CCO and APCO data were scaled using a UFS datum in the beginning of each experiment as a reference value. The mean values of the ABP, CVP and PAP data are shown in yellow. The APCO data may not be accurate due to the fact that the APCO method is not reliable in animals.

Figure 2a is an example of measured data from the pig #9 in the first animal study, displaying CO, SV, CO_Scaled_, SV_Scaled_, HR, arterial blood pressure (ABP), central venous pressure (CVP) and pulmonary artery pressure (PAP) during the experiment. In the CO_Scaled_ and SV_Scaled_ plots of Fig. 2a, the EIT and APCO data were scaled using a PAC-CCO datum at the beginning of each experiment as a reference value after a time-delay adjustment was applied to the PAC-CCO data. Figure 2b shows an example of measured data from the pig #27 in the second animal study, showing CO, SV, CO_Scaled_, SV_Scaled_, HR, ABP, CVP and PAP data. In the CO_Scaled_ and SV_Scaled_ plots of Fig. 2b, the EIT, PAC-CCO and APCO data were scaled using a UFS datum in the beginning of each experiment as a reference value.

Compared with the ABP data, the CO_PAC-CCO_ data in both Fig. 2a,b show a noticeable time delay due to HemoSphere-CCO’s internal data averaging and processing before outputting CO_PAC-CCO_. The time delay in CO_PAC-CCO_ was estimated and adjusted as described earlier. The estimated time delay in CO_PAC-CCO_ from all 23 pigs ranged from 4.7 to 11.1 min, which is consistent with the known internal time delay of the HemoSphere-CCO in its CO measurements of 5 to 12 min^49^. However, this time-delay adjustment method could lead to some error if and when the time delay in the PAC-CCO device varies during the experiment. Sections S7 and S8 of the online supplementary materials show the data acquired throughout each experiment for all 23 pigs.

**Figure 3:**
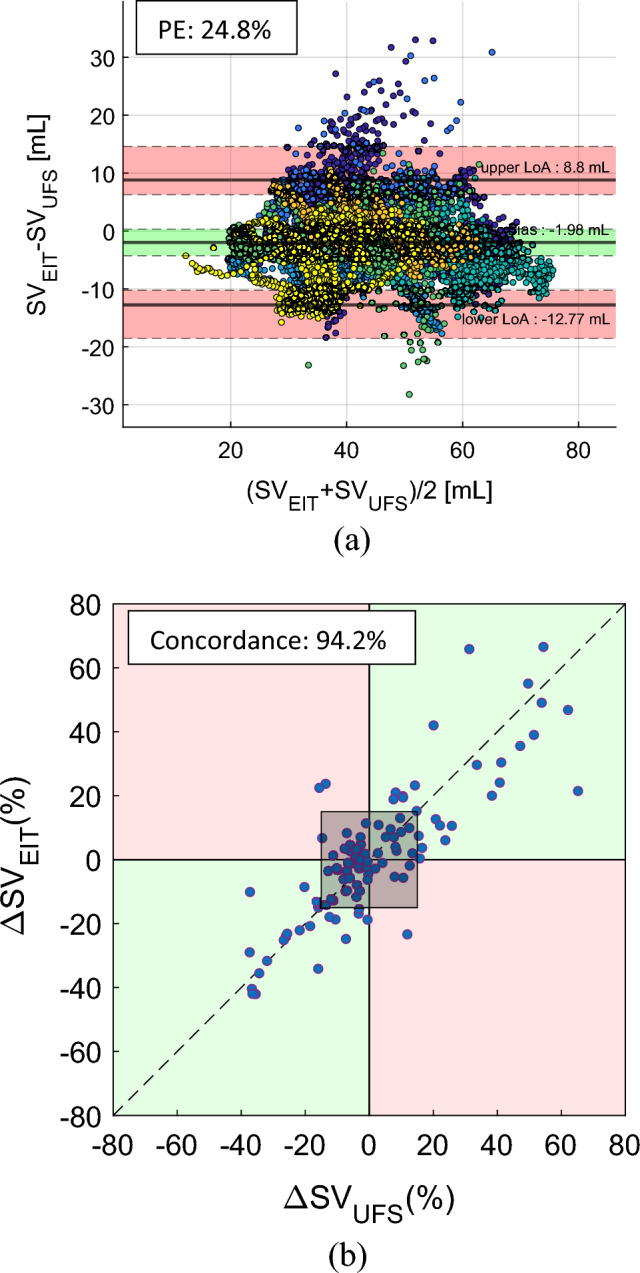
(**a**) Bland–Altman plot between SV_EIT_ and SV_UFS_ using the pooled data from 7 pigs (pigs #22– #32 excluding pigs #22, #24, #29 and #32). The percentage error (PE) between SV_EIT_ and SV_UFS_ in the pooled data from 7 pigs was 24.8%. (**b**) Plot for 10-min concordance of 94.2% between SV_EIT_ and SV_UFS_ using the pooled data from 7 pigs (pigs #22– #32 excluding pigs #22, #24, #29 and #32). The shaded square at the center of the plot is the 15% exclusion band.

Figure 3a shows the Bland–Altman plot between SV_UFS_ and SV_EIT_ using 12,360 data pairs pooled together from 7 pigs. The bias was − 1.98 mL with the 95% CI of (− 4.29, 0.32) mL. The 95% LoA were − 12.8 and 8.80 mL with the 95% CI of (− 18.6, 14.6) mL. The overall PE between SV_UFS_ and SV_EIT_ was 24.8%, while the PE for individual pigs ranged from 14.0 to 29.9%. As shown in Fig. 3b, the 10-min concordance between SV_UFS_ and SV_EIT_ was 94.2% using 104 pairs of data pooled together from 7 pigs.

**Figure 4:**
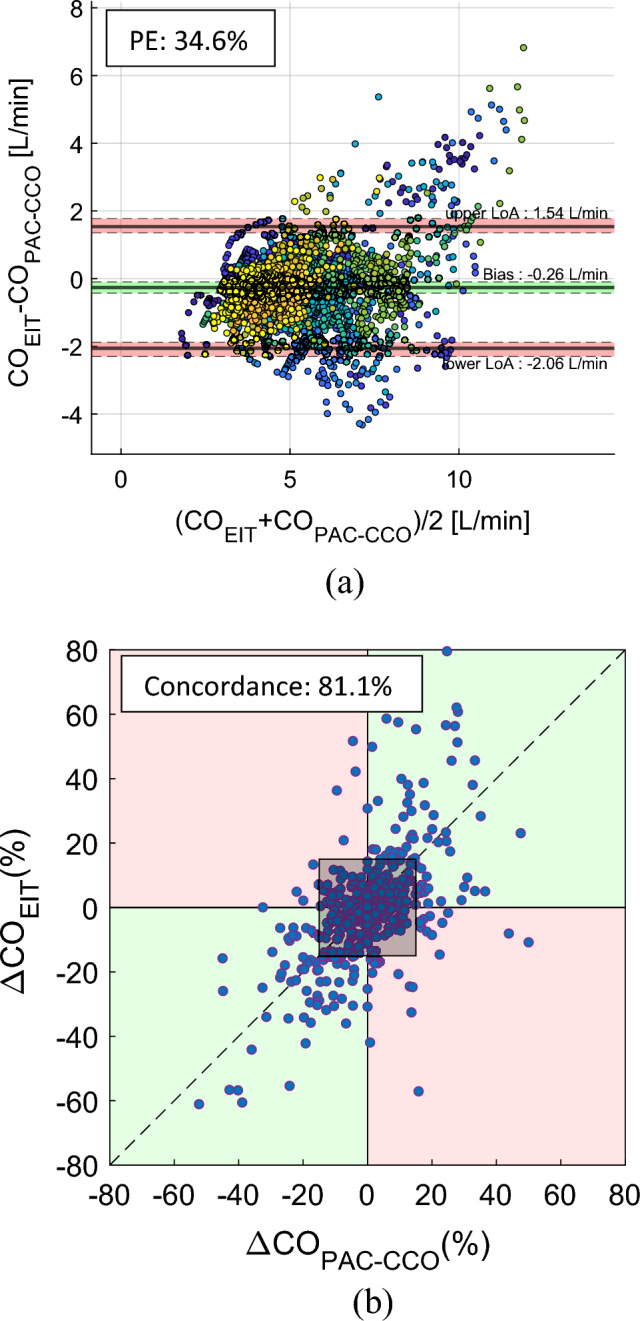
(**a**) Bland–Altman plot between CO_EIT_ and time-delay-adjusted CO_PAC-CCO_ using the pooled data from 23 pigs (pigs #5–#32 excluding pigs #10, #22, #24, #29 and #32). The percentage error (PE) between CO_EIT_ and time-delay-adjusted CO_PAC-CCO_ in the pooled data from 23 pigs was 34.6%. (**b**) Plot for 10-min concordance of 81.1% between CO_EIT_ and time-delay-adjusted CO_PAC-CCO_ using the pooled data from 23 pigs (pigs #5–#32 excluding pigs #10, #22, #24, #29 and #32). The shaded square at the center of the plot is the 15% exclusion band.

Figure 4a shows the Bland–Altman plot between CO_EIT_ and time-delay-adjusted CO_PAC-CCO_ using 4,676 data pairs pooled together from 23 pigs. The bias was − 0.26 L/min with the 95% CI of (− 0.43, − 0.10) L/min. The 95% LoA were − 2.06 and 1.54 L/min with the 95% CI of (− 2.30, 1.77) L/min. The overall PE was 34.6%, while the PE for individual pigs ranged from 15.2 to 47.8%. Figure 4b shows the 10-min concordance between CO_EIT_ and time-delay-adjusted CO_PAC-CCO_ was 81.1% using 451 pairs of data pooled together from 23 pigs.

Table 1 summarizes the results of all Bland–Altman and concordance analyses for all 12 pairs of measured data, i.e., $\left({{\text{CO}}}_{{\text{UFS}}},{{\text{CO}}}_{{\text{EIT}}}\right)$, $\left({{\text{CO}}}_{{\text{UFS}}},{{\text{CO}}}_{{\text{PAC}}-{\text{CCO}}}\right)$, $\left({{\text{CO}}}_{{\text{UFS}}},{{\text{CO}}}_{{\text{APCO}}}\right)$, $\left({{\text{SV}}}_{{\text{UFS}}},{{\text{SV}}}_{{\text{EIT}}}\right)$, $\left({{\text{SV}}}_{{\text{UFS}}},{{\text{SV}}}_{{\text{PAC}}-{\text{CCO}}}\right)$, $\left({{\text{SV}}}_{{\text{UFS}}},{{\text{SV}}}_{{\text{APCO}}}\right)$, $\left({{\text{CO}}}_{{\text{PAC}}-{\text{CCO}}},{{\text{CO}}}_{{\text{EIT}}}\right)$, $\left({{\text{CO}}}_{{\text{PAC}}-{\text{CCO}}},{{\text{CO}}}_{{\text{APCO}}}\right)$, $\left({{\text{CO}}}_{{\text{APCO}}},{{\text{CO}}}_{{\text{EIT}}}\right)$, $\left({{\text{SV}}}_{{\text{PAC}}-{\text{CCO}}},{{\text{SV}}}_{{\text{EIT}}}\right)$, $\left({{\text{SV}}}_{{\text{PAC}}-{\text{CCO}}},{{\text{SV}}}_{{\text{APCO}}}\right)$ and $\left({{\text{SV}}}_{{\text{APCO}}},{{\text{SV}}}_{{\text{EIT}}}\right)$. For ${{\text{CO}}}_{{\text{PAC}}-{\text{CCO}}}$ and ${{\text{SV}}}_{{\text{PAC}}-{\text{CCO}}}$ each, two cases with and without time-delay adjustment are included in Table 1. The concordance analyses were conducted at intervals of 1 min, 5 min and 10 min in Table 1. The complete results of the Bland–Altman and concordance analyses are included in Tables S2 and S3 of the online supplementary materials (Section S9).

**Table 1:** Percentage error (PE) and concordance between data pairs measured by using a reference device (${{\text{X}}}_{{\text{REF}}}$) and device under test (${{\text{X}}}_{{\text{DUT}}}$).

Data pairs		PE (%)	Concordance (%)			Sources of data
${{\text{X}}}_{{\text{REF}}}$	${{\text{X}}}_{{\text{DUT}}}$		1 min	5 min	10 min	
${{\text{CO}}}_{{\text{UFS}}}$	${{\text{CO}}}_{{\text{EIT}}}$	26.1	74.4	88.8	92.5	Study #2 (7 pigs)
	${{\text{CO}}}_{{\text{PAC}}-{\text{CCO}}}$	37.9	27.8	41.1	51.8	
	${{\text{CO}}}_{{\text{PAC}}-{\text{CCO}}}$(time delay adjusted)	22.7	68.2	94.5	92.7	
	${{\text{CO}}}_{{\text{APCO}}}$	33.1	74.4	62.2	69.8	
${{\text{SV}}}_{{\text{UFS}}}$	${{\text{SV}}}_{{\text{EIT}}}$	24.8	80.7	88.7	94.2	
	${{\text{SV}}}_{{\text{PAC}}-{\text{CCO}}}$	34.8	52.9	61.2	66.1	
	${{\text{SV}}}_{{\text{PAC}}-{\text{CCO}}}$(time delay adjusted)	24.2	65.5	86.4	91.3	
	${{\text{SV}}}_{{\text{APCO}}}$	34.5	55.1	60.8	71.7	
${{\text{CO}}}_{{\text{PAC}}-{\text{CCO}}}$	${{\text{CO}}}_{{\text{EIT}}}$	44.9	32.0	40.9	58.3	Study #1 and #2 (23 pigs)
	${{\text{CO}}}_{{\text{APCO}}}$	45.8	32.6	46.0	54.1	
${{\text{CO}}}_{{\text{PAC}}-{\text{CCO}}}$(time delay adjusted)	${{\text{CO}}}_{{\text{EIT}}}$	34.6	68.6	78.9	81.1	
	${{\text{CO}}}_{{\text{APCO}}}$	34.8	71.0	69.7	73.3	
${{\text{CO}}}_{{\text{APCO}}}$	${{\text{CO}}}_{{\text{EIT}}}$	42.5	64.0	70.4	73.1	
${{\text{SV}}}_{{\text{PAC}}-{\text{CCO}}}$	SV_EIT_	40.6	45.6	46.1	50.5	
	SV_APCO_	40.9	50.0	55.0	57.9	
${{\text{SV}}}_{{\text{PAC}}-{\text{CCO}}}$(time delay adjusted)	SV_EIT_	31.4	51.0	68.9	72.4	
	SV_APCO_	32.9	55.7	62.4	66.3	
SV_APCO_	SV_EIT_	38.8	53.2	66.7	69.3	

Details about the Bland–Altman and concordance analyses are described in the online supplementary materials (Section S9). The results for the APCO method should not be interpreted conclusively since the APCO method is not reliable on animals.

## Discussion

We designed the animal studies described in this paper to effect changes in cardiac preload, afterload and contractility conditions. To evaluate the performance of the EIT device over a wide range of CO and SV, we used pharmacological interventions to vary afterload and contractility conditions, while preload conditions were varied by bleeding and volume resuscitation.

### Hemodynamic responses to interventions

For all 23 pigs, the administration of vasodilator (nitroprusside) and vasopressor (phenylephrine) decreased and increased ABP, respectively. However, the changes in SV in response to the vasodilator and vasopressor varied depending on the volume status of each animal^50^. When the amount of stressed volume^51,52^ was large, the administration of nitroprusside (phenylephrine) decreased (increased) the afterload, which led to larger (smaller) SV, as observed in pigs #8, #12, #15, #17 and #21 (see Sections S7 and S8 in the online supplementary materials). On the other hand, in animals with a small amount of stressed volume and thereby already-reduced venous return, the administration of nitroprusside (phenylephrine) decreased (increased) the preload through decreased (increased) venous return, which led to smaller (larger) SV, as observed in pigs #5, #7, #9, #13, #19, #20, #23, #25, #26, #27, #28 and #31. In pigs #6, #11, #14, #16, #18 and #30, the changes in SV after the administration of nitroprusside and phenylephrine either were very small or did not exhibit a causal relationship.

For all 7 pigs in the second animal study, the administration of pulmonary artery vasopressor (thromboxane) increased PAP^53,54^, resulting in increased afterload of the right ventricle. In pigs #23, #27, #28 and #30, SV decreased due to the increased right ventricular afterload. However, SV did not decrease in pigs #25, #26 and #31, most likely due to the Anrep effect to increase the contractility of the right ventricle in response to the increased PAP^55,56^.

In all 23 pigs, the administration of the positive and negative inotrope (dobutamine and esmolol) increased and decreased CO, respectively. However, the changes in SV after the administration of the inotrope varied depending on animals. In pigs #12, #14, #15, #16, #17, #18, #21, #23, #30 and #31, the changes in SV followed those of CO. In other pigs, SV either decreased or did not increase since the increased HR in response to dobutamine shortened the ventricular filling time.

In most pigs, bleeding reduced the stressed volume and thereby the preload, leading to decreased SV, while volume resuscitation restored the stressed volume to increase the venous return and thereby increased SV. When fluid was used for volume resuscitation, the stressed volume should have increased initially and then gradually decreased as the fluid moved out of the intravascular space^57^. When blood was used for volume resuscitation, the stressed volume should have increased and remained at this increased volume. However, in our studies where fluid or blood was administered slowly and continuously, we were not able to observe the differences between fluid and blood in the measured SV changes. The above observations are based on visual analysis of the plots. More in-depth quantitative analyses with more subjects would be useful in the future.

### Performance comparison of EIT with UFS, PAC-CCO and APCO

For vascular tone estimation, the APCO method uses a patient’s age, gender, height, weight, body surface area, and the shape of the arterial pressure waveform that depends on the geometry of the arterial vasculature and its branching. Since it was developed for human subjects and the APCO data acquired from the pigs may contain large errors^58,59^, the results associated with the acquired APCO data in the current study could not be discussed quantitatively and interpreted conclusively.

In Fig. 2b and also in Section S8 of the online supplementary materials, we can see that the patterns of CO_EIT_ and SV_EIT_ over time match well with those of CO_UFS_ and SV_UFS_ in all 7 pigs. On the other hand, the timecourses of CO_PAC-CCO_ appear to be much delayed and smoothed out compared with those of CO_UFS_. This suggests that PAC-CCO would be useful in measuring CO when it does not change much. The timecourses of CO_APCO_ and SV_APCO_ match relatively well with those of CO_UFS_ and SV_UFS_. However, there are some discrepancies from time to time due to the limitations of the APCO method when applied to animals.

In Table 1, the PE value was 26.1% between CO_UFS_ and CO_EIT_, and it was 24.2% between SV_UFS_ and SV_EIT_, which meets the acceptance criteria for interchangeability with PAC-ICO. The PE was 22.7% between the time-delay-adjusted CO_PAC-CCO_ and CO_UFS_. Although this meets the acceptance criteria for interchangeability, the time delay was estimated in post-processing and it is not available to clinicians in practice. Without time-delay adjustments, the PE between CO_PAC-CCO_ and CO_UFS_ increased to 44.9%. On the other hand, there was a negligible time delay in CO_EIT_ and SV_EIT_. The PE between CO_EIT_ and time-delay-adjusted CO_PAC-CCO_ was 34.6% over a wide range of cardiac output (2.49–14.6 L/min). This indicates that the EIT device would be comparable with the time-delay-adjusted PAC-CCO device in measuring CO.

### Pros and cons of PAC-CCO, APCO and EIT

Although PAC-CCO has been used in place of PAC-ICO primarily due to its convenience, it is still a very invasive clinical method with well-known side effects^5,6^. Due to its inherent time delay of 5 to 12 min, PAC-CCO is inappropriate for dynamic hemodynamic monitoring, e.g., fluid responsiveness test. Although APCO is less invasive compared with the PAC methods, it is still invasive requiring arterial catheterization.

In contrast to the conventional impedance cardiography with single or dual channels having experienced limited clinical acceptance^15–19^, the EIT device used in this study was designed to measure CO and SV with a much larger number of impedance-measurement channels. Utilizing 208 channels of impedance data, the EIT device could robustly extract the component of small cardiogenic impedance changes using the recently-developed leadforming method^31^.

In our study, the EIT device exhibited promising performance compared with that of UFS and PAC-CCO. Once its performance is confirmed on patients in future clinical studies, the EIT device could become a clinically-useful noninvasive hemodynamic monitor on selected patients, especially for dynamic hemodynamic monitoring.

Since the EIT technology has been clinically used for regional lung ventilation imaging, it would make sense to combine such ventilation imaging functionalities with those of hemodynamic monitoring in the future. On a mechanically-ventilated patient, titration of positive end-expiratory pressure (PEEP) and fluid administration are often needed, preferably on an individual basis. While EIT has been used for personalized PEEP titration, separate hemodynamic monitors have been used for personalized fluid management. Since different mechanical ventilation and fluid management strategies result in complicated and patient-dependent heart–lung interactions affecting the lungs and distal organs, it would be ideal if one integrated device is used for both decisions. If EIT’s hemodynamic monitoring functions are validated and combined with its ventilation imaging functions, it could potentially become a key cardiopulmonary monitoring and decision-supporting device in ICU, especially for simultaneously- and/or separately-performed personalized PEEP titration and fluid management. However, it might be difficult to use the EIT device on extremely obese patients, and the EIT device due to its use of surface electrodes would be vulnerable to ESU artefacts and large body movements during surgery in OR. For the same reason, it may not be possible to use EIT on patients during high abdominal and thoracic surgeries.

## Limitations

Although 11 pigs were used in the second study including UFS around the pulmonary artery via thoracotomy, we were able to use the data from only 7 pigs in the statistical analyses. In comparison of EIT with UFS, the 95% CIs should have been narrower if the number of pigs in the second study were increased. However, compared with other studies where PAC-ICO was used as a reference method, the number of data points in our study (a total of 12,360 data pairs) was much larger since both UFS and EIT provide measurements at every 5 s while PAC-ICO can produce only intermittent results.

We tried to synchronize all the acquired data from the multiple devices as accurately as possible. However, there could have been some differences in time alignments among the devices on the results of the Bland–Altman analyses. Although the EIT and UFS data were time-aligned to each other much better than the PAC-CCO and APCO data, we believe that the influences of time misalignments were not large enough to change our conclusions.

Although the PAC-ICO method is the current clinical gold standard^40–43^, we could not use it in this study since the HemoSphere-ICO was not available in Korea. Lamia et al.^60^ used PAC-ICO and four other methods to measure CO on 21 patients in ICU. For all 10 combinations of the five methods, the PE ranged from 33 to 59%. The PE between PAC-ICO and impedance cardiography was 47%. Joosten et al.^36^ found that six commercially-available impedance cardiography devices have a pooled-estimate PE of 42% compared to PAC-ICO. We expect the PE between CO_EIT_ and CO_PAC-ICO_ would be lower than that (34.6% in our study) between CO_EIT_ and time-delay-adjusted CO_PAC-CCO_ since PAC-ICO is more accurate than PAC-CCO.

The accuracy (bias in the Bland–Altman analysis) of the measured CO and SV data was not evaluated in this paper since we minimized bias values using the volume calibration method described earlier to convert relative SV_EIT_ to absolute SV_EIT_. The LoA and PE values presented in this paper, therefore, represent the best performance of each device. As suggested by da Silva Ramos et al.^28^, we also believe that a subject-specific volume calibration using anthropometric data, such as weight and height, is needed to convert relative SV_EIT_ to absolute SV_EIT_ in mL. This requires clinical studies on normal human subjects and patients to acquire training data sets including simultaneously-measured data using the EIT device and a clinical gold standard reference device.

## Conclusion

Based on the results from two animal studies on 23 pigs (PAC-CCO and APCO were used on all 23 while UFS was additionally used on 7 of them), the precision of the EIT device in measuring CO and SV was found to be comparable to that of the UFS placed around the pulmonary artery and the commercially-available invasive hemodynamic monitor using the PAC-CCO method. Although we assessed the feasibility of SV/CO measurements using the proposed EIT method, the method needs to be thoroughly validated on human subjects through future clinical studies with real-world clinical scenarios. Future clinical studies would be needed to evaluate the EIT’s accuracy and precision and assess its potential usefulness in various clinical settings and patient populations.

### Supplementary Information

Supplementary Information.

## Data Availability

The data that support the findings of this study are available from the corresponding author, EJW, upon reasonable request.

## Abbreviations

ABGA: Arterial blood gas analysis
ABP: Arterial blood pressure
APCO: Arterial pressure-based cardiac output
CI: Confidence interval
CO: Cardiac output
CVP: Central venous pressure
DUT: Device under test
EIT: Electrical impedance tomography
ESU: Electrosurgical unit
PEEP: Positive end-expiratory pressure
HR: Heart rate
ICU: Intensive care unit
LoA: Limits of agreement
MAP: Mean arterial pressure
OR: Operating room
PAC: Pulmonary artery catheterization
PAC-CCO: Pulmonary artery catheterization-continuous cardiac output
PAC-ICO: Pulmonary artery catheterization-intermittent cardiac output
PAP: Pulmonary artery pressure
PCA: Pulse contour analysis
PE: Percentage error
REF: Reference device
SV: Stroke volume
TPTD: Transpulmonary thermodilution
UFS: Ultrasonic flow sensor
