# Supplementary material for: Comparison of noninvasive cardiac output and stroke volume measurements using electrical impedance tomography with invasive methods in a swine model
Source: Sci Rep. 2024 Feb 5;14:2962. doi: 10.1038/s41598-024-53488-0 (PMC10844629; doi:10.1038/s41598-024-53488-0)
Supplement: Supplementary file 1 — Supplementary Information. [file 41598_2024_53488_MOESM1_ESM.pdf]

Supplementary Materials

Comparison of noninvasive cardiac output and stroke volume measurements using electrical impedance tomography with invasive methods in a swine Model

Chi Ryang Chung[1], Ryoung Eun Ko[1], Geuk Young Jang[2], Kyounghun Lee[2], Gee Young Suh[1], Yongmin Kim[3] and Eung Je Woo[2]

[1]Department of Critical Care Medicine, Samsung Medical Center, Sungkyunkwan University School of Medicine, Seoul, Korea

[2]Department of Biomedical Engineering, Kyung Hee University, Seoul, Korea

[3]Department of Convergence IT Engineering, POSTECH, Pohang, Korea

Corresponding author:

Eung Je Woo, Professor

Department of Biomedical Engineering, College of Medicine, Kyung Hee University

26 Kyungheedae-ro, Dongdaemun-gu, Seoul 02447, Korea

Phone: +82-2-961-9502, Fax: +82-2-958-2889, Email: ejwoo@khu.ac.kr

S1. Protocol of the first study

We began each experiment with a baseline/stabilization part where MAP was stably maintained for at least 5 minutes. This baseline/stabilization part was repeated after each intervention. The part #1 was to measure CO and SV under different afterload conditions using the following protocol:

1. Nitroprusside was slowly administered while increasing its dose until MAP decreased to 60 mmHg. The dose was maintained for 15 minutes.
2. Waited until MAP returned to 70~80 mmHg. Further waited for 5 minutes.
3. Phenylephrine was slowly administered while increasing its dose until MAP increased above 85 mmHg. The dose was maintained for 15 minutes.
4. Waited until MAP returned to 70~80 mmHg. Further waited for 5 minutes.

The part #2 was to measure CO and SV under different contractility conditions as follows:

1. Dobutamine was slowly administered while increasing its dose until MAP increased above 100 mmHg. The dose was maintained for 5 minutes.
2. Waited until MAP returned to 70~80 mmHg. When MAP did not return to 70~80 mmHg, crystalloid fluid (Plasma Solution-A Injection, CJ Healthcare, Korea) was administered until MAP returned to 70~80 mmHg. Waited for 5 minutes.
3. Esmolol was slowly administered while increasing its dose until MAP decreased below 60 mmHg. The dose was maintained for 15 minutes.
4. Waited until MAP returned to 70~80 mmHg for 30 minutes.

The part #3 was to measure CO and SV under different preload conditions as follows:

1. Blood was withdrawn from the animal through a needle, which was inserted into a blood vessel and connected to a blood bag, until MAP decreased below 50 mmHg. The amount of blood withdrawn and the bleeding rate varied in different animals.
2. Waited for 5 minutes without withdrawing additional blood.
3. 1 L of crystalloid fluid (Plasma Solution-A Injection, CJ Healthcare, Korea) was intravenously administered for 20 minutes. When MAP decreased below 50 mmHg during fluid administration, dobutamine was administered until MAP increased above 50 mmHg.
4. Waited for about 20 minutes.

S2. Protocol of the second study

We began each experiment with a baseline/stabilization part where MAP was stably maintained for at least 5 minutes. This baseline/stabilization part was repeated after each intervention. The part #1 was the same as the part #1 of the first study.

The part #2 was the same as the part #2 of the first study.

The part #3 was to measure CO and SV under different afterload conditions using the following protocol:

1. Thromboxane was slowly administered while increasing its dose until the mean PAP increased to 35 mmHg. The dose was maintained for 5 minutes.
2. Waited until the mean PAP returned to 25 mmHg. Further waited for 5 minutes.

The part #4 was to measure CO and SV under different preload conditions as follows:

1. Blood was withdrawn from the animal through a needle, which was inserted into a blood vessel and connected to a blood bag, until MAP decreased below 50 mmHg. The total amount of blood withdrawn and the bleeding rate varied in different animals.
2. Waited for 5 minutes without withdrawing additional blood.
3. The removed blood was transfused into the pig for 5 to 10 minutes. When MAP decreased below 50 mmHg during fluid administration, dobutamine was administered until MAP increased above 50 mmHg.
4. Waited for about 20 minutes.

S3. Thoracotomy procedure

Each pig was placed on an operating table in the right lateral position. Thoracotomy started at the 5th or 6th intercostal space. Subcutaneous tissues, muscles and pleura were dissected using an electrosurgical unit (ESU). Then, a retractor was placed between the ribs to secure a view to install the C-shaped UFS around the pulmonary artery while the pericardium was open. There was little displacement of the heart during this thoracotomy procedure. After checking the signal from the installed UFS around the pulmonary artery, the dissected layers were sutured and wound areas were taped to close the chest. Then, we attached 16 sensing electrodes and 1 reference electrode for EIT measurements around the chest. We did not observe any noticeable changes in the acquired signals before and after the thoracotomy procedure. We believe that the thoracotomy did not affect the PAC-CCO, APCO and EIT measurements per se.

S4. EIT data collection method

The EIT device injected current between a chosen neighboring electrode pair. For each current injection, 16 voltage data were measured simultaneously (parallel measurements). 13 of them were used for SV/CO calculations and 3 were used to estimate electrode-skin contact impedance values. This was repeated for all 16 neighboring current-injecting electrode pairs in 10 ms. Therefore, for every 10 ms, 13×16 (208) impedance data were measured for SV/CO calculations. Fig. S1(a) shows an example of the EIT device's 208-channel impedance signals from an animal over a time period of 20 seconds. In plotting Fig. S1(a), their dc offsets were removed, and the amplitudes were normalized for better visualization. The impedance signals show small and fast fluctuations representing cardiogenic components originating from blood volume changes as well as large and slow fluctuations representing ventilatory components originating from air volume changes. In Fig. S1(b), 13 impedance signals $z_{8,1}$ to $z_{8,13}$ enclosed in the red box in (a) are plotted with artificially-added dc offsets to visualize all 13 waveforms clearly. On the right side of Fig. S1(a), the weight vector $\mathbf{w} = [w_1, w_2, \cdots, w_{208}]^{\mathrm{T}}$ is plotted, which was computed using the recently-developed leadforming algorithm[25]. The EIT device produced the cardiac volume signal (CVS) in real time shown in Fig. S2(a) by applying this weight vector to the 208-channel impedance signals. Fig. S2(b) shows the power spectrum of the CVS in Fig. S2(a) where the fundamental frequency of the cardiogenic component is about 1.2 Hz. Beat-to-beat SV values were first derived from the extracted CVS. Then, an average SV value during the most-recent 5-second interval was computed, displayed and stored every 5 second.

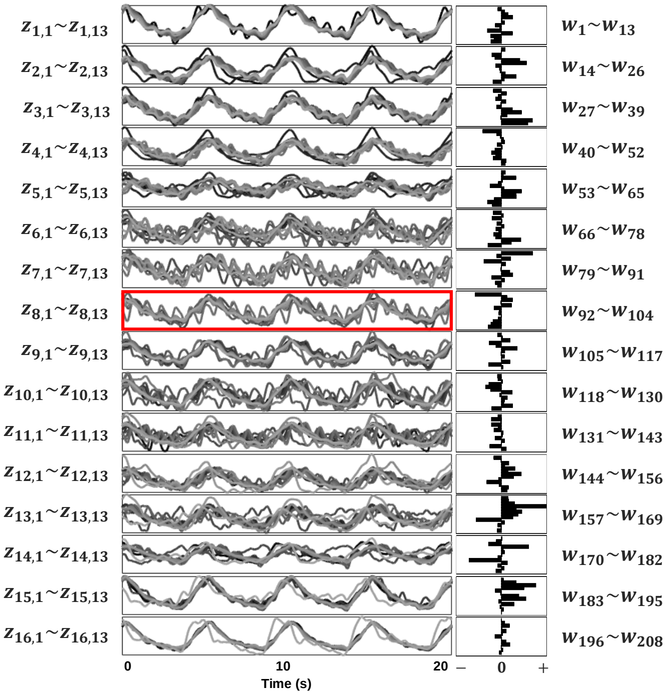

(a)

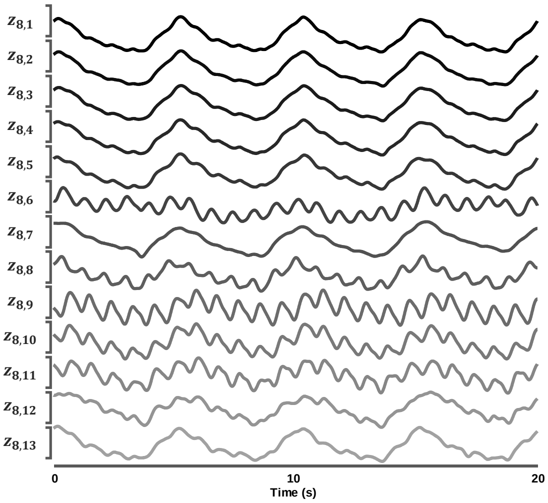

(b)

Fig. S1. (a) 208-channel normalized impedance signals measured by the EIT device showing large/slow variations associated with lung ventilation and small/fast fluctuations associated with cardiac blood flow. The weight vector $\mathbf{w}_{\mathrm{B}}$ described by Lee *et al.*[25] is plotted on the right side. (b) Enlarged view of 13 voltage signals enclosed in the red box in (a) with artificially-added dc offsets for clear visualization.

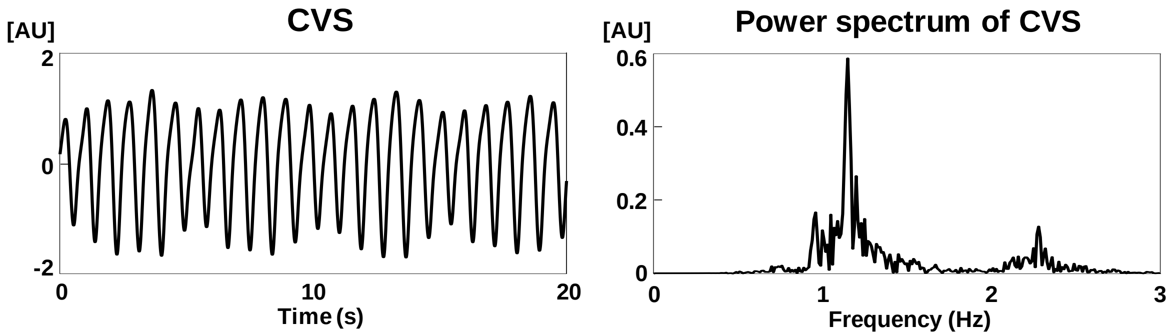

(a) (b)

Fig. S2. (a) Example of the CVS extracted from the 208-channel impedance data shown in Fig. S1 using the leadforming algorithm[25]. (b) Power spectrum of the CVS in (a) shows a fundamental frequency of about 1.2 Hz corresponding to a heart rate of about 72 bpm.

S5. Percentage error and concordance

To compute the percentage error (PE), we let $\mathrm{X}_{i,j}^{REF}$ and $\mathrm{X}_{i,j}^{DUT}$ be the *j*th data from the *i*th animal using the reference device and the device under test, respectively. The number of data pairs from the ith animal is m_i and the number of animals is n. Then, the total number of data pairs is $N = \sum_{i=1}^{n} m_i$. Denoting the difference as $d_{i,j} \coloneqq \mathrm{X}_{i,j}^{DUT} - \mathrm{X}_{i,j}^{REF}$, we compute the PE as follows:

$$\mathrm{PE} = 1.96 \times \frac{\hat{\sigma}_d}{\hat{\mu}} \times 100$$

where

$$\hat{\mu} = \frac{1}{n}\sum_{i=1}^{n}\frac{1}{m_i}\sum_{j=1}^{m_i}\frac{\mathrm{X}_{i,j}^{DUT} + \mathrm{X}_{i,j}^{REF}}{2} \quad \text{and}$$

$$\hat{\sigma}_d = \sqrt{\frac{1}{n-1}\sum_{i}\left(\bar{d}_i - \bar{d}\right)^2 + \left(1 - \frac{1}{m_h}\right)\frac{1}{N-n}\sum_{i,j}\left(d_{i,j} - \bar{d}_i\right)^2}$$

with $\bar{d}_i = \frac{1}{m_i}\sum_{j=1}^{m_i} d_{i,j}$, $\bar{d} = \frac{1}{n}\sum_{i=1}^{n} \bar{d}_i$ and m_h is the harmonic mean of $\{m_i\}_{i=1}^{n}$. Here, $\hat{\sigma}_d$ is the standard deviation of the difference accounting for inter-subject biases.

The condition of concordance in measured data $\mathrm{X}_{\mathrm{DEV1}}$ and $\mathrm{X}_{\mathrm{DEV2}}$ by the DEV1 and DEV2 was defined as follows:

$$\Delta\mathrm{X}_{\mathrm{DEV1}}(i) \cdot \Delta\mathrm{X}_{\mathrm{DEV2}}(i) > 0$$

where $\Delta\mathrm{X}_{\mathrm{DEV1}}(i)$ and $\Delta\mathrm{X}_{\mathrm{DEV2}}(i)$ are the percentage difference in $\mathrm{X}_{\mathrm{DEV1}}$ and $\mathrm{X}_{\mathrm{DEV2}}$ at time *i*, respectively, as follows:

$$\Delta\mathrm{X}_{\mathrm{DEV1}}(i) = \frac{\mathrm{X}_{\mathrm{DEV1}}(i+1) - \mathrm{X}_{\mathrm{DEV1}}(i)}{\mathrm{X}_{\mathrm{DEV1}}(i)} \times 100(\%)$$

and

$$\Delta X_{\mathrm{DEV2}}(i) = \frac{X_{\mathrm{DEV2}}(i+1) - X_{\mathrm{DEV2}}(i)}{X_{\mathrm{DEV2}}(i)} \times 100(\%)$$

where $(i+1)$ and i are two consecutive time points when the entire experiment time was divided into non-overlapping 10-min intervals. Similarly, the condition of discordance in measurements X was defined as

$$\Delta X_{\mathrm{DEV1}}(i) \cdot \Delta X_{\mathrm{DEV2}}(i) < 0.$$

The concordance of X denoted as $\mathrm{CCD}_{\mathrm{X}}$ was computed as the percentage of data pairs satisfying the concordance condition outside an exclusion band of 15% in both ΔX_{DEV1} and ΔX_{DEV2} as follows:

$$\mathrm{CCD}_{\mathrm{X}} = \frac{\#(A_{CCD} \setminus A_{EXD})}{\#((A_{CCD} \cup A_{DCD}) \setminus A_{EXD})} \times 100(\%)$$

where $\#A$ denotes the number of elements contained in the set A, A_{EXD} is the set of data pairs in the exclusion band, A_{CCD} is the set of data pairs satisfying the concordance condition, and A_{DCD} is the set of data pairs satisfying the discordance condition. The sets were defined as

$$A_{EXD} = \{i : |\Delta X_{\mathrm{DEV1}}(i)| < 15 \text{ and } |\Delta X_{\mathrm{DEV2}}(i)| < 15\},$$

$$A_{CCD} = \{i : \Delta X_{\mathrm{DEV1}}(i) \cdot \Delta X_{\mathrm{DEV2}}(i) > 0\} \text{ and}$$

$$A_{DCD} = \{i : \Delta X_{\mathrm{DEV1}}(i) \cdot \Delta X_{\mathrm{DEV2}}(i) < 0\}.$$

S6. Information about 32 animals in the first and second study

Table S1. Information about the 32 animal experiments. Pigs #1~ #4, #10, #22, #24, #29 and #32 were excluded in statistical analyses. The ABGA results were from the first blood sample taken at the beginning of the part #1.

No.	Weight (kg)	ABGA				Final Dosage					Bleeding	
		pH	pO_2 (mmHg)	pCO_2 (mmHg)	HCO_3^- (mmol/L)	Nitroprusside (μg/kg/min)	Phenylephrine (μg/kg/min)	Dobutamine (μg/kg/min)	Esmolol (μg/kg/min)	Thromboxane (μg/kg/min)	Volume (mL)	Rate (mL/min)
1	*63.0*	*7.43*	*507*	*51.5*	*34.2*	Not used	*1.37*	*18.00*	*500*	-	*985*	-
2	*68.3*	*7.42*	*496*	*39.7*	*25.8*	*3.40*	*0.59*	*12.20*	*439*	-	*1000*	-
3	*77.0*	-	-	-	-	-	-	-	-	-	-	-
4	*68.3*	*7.40*	*524*	*66.0*	*40.9*	*2.37*	*0.78*	*10.74*	*146*	-	*600*	-
5	65.5	7.44	369	49.0	33.8	1.22	1.17	11.20	293	-	1150	23.7
6	70.3	7.42	500	57.6	38.2	3.42	0.87	5.69	341	-	1150	29.6
7	65.5	7.45	482	46.6	32.8	1.22	0.35	10.18	242	-	800	26.6
8	64.6	7.47	519	48.2	35.3	1.46	1.17	5.16	645	-	690	30.2
9	69.3	7.48	486	49.8	37.7	2.20	0.3	5.29	500	-	600	48.8
10	*70.4*	*7.45*	*303*	*47.0*	*33.4*	*0.98*	*0.98*	*7.32*	*293*	-	*900*	-
11	66.4	7.42	471	51.1	33.3	0.73	0.68	7.32	293	-	700	47.4
12	70.9	7.43	420	54.8	36.8	1.22	0.78	7.32	846	-	1000	36.5
13	65.1	7.43	501	45.1	30.0	0.98	0.78	7.32	768	-	800	48.9
14	73.2	7.49	515	48.7	37.9	1.22	0.59	3.64	439	-	700	47.5
15	66.2	7.48	529	44.7	33.4	0.73	1.37	4.73	504	-	500	43.6
16	67.0	7.47	439	49.7	36.6	0.98	0.59	4.73	572	-	800	48.2
17	61.3	7.42	486	49.2	32.5	0.54	0.68	3.26	544	-	400	54.9
18	69.6	7.48	407	52.7	39.3	3.17	1.17	3.35	1197	-	550	49.7
19	67.1	7.48	352	39.8	29.7	0.73	2.48	7.32	98	-	900	52.8
20	70.4	7.50	468	43.1	34.2	1.22	0.98	4.73	379	-	400	46.7
21	70.0	7.46	441	51.2	37.2	0.73	0.78	4.73	293	-	800	43.1
22	67	7.41	425	44.8	28.5	1.22	0.85	9.76	149	0.120	900	-
23	64	7.47	564	49.2	35.9	2.6	0.73	5.21	520	0.063	600	28.8
24	68	-	-	-	-	-	-	-	-	-	-	-
25	64	7.41	257	49.6	31.1	1.04	Not used	4.69	1302	0.07	500	90.4
26	68	7.41	291	44.0	27.9	0.74	0.78	9.80	980	0.10	300	115.4
27	74	7.47	326	41.5	30.4	Not used	0.81	9.01	Not used	0.15	350	99.5
28	68	7.47	566	39.5	28.5	3.43	0.29	9.80	1078	0.12	550	99.4
29	69	-	-	-	-	-	-	-	-	-	-	-
30	67	7.52	496	36.2	29.4	Not used	0.70	Not used	Not used	0.090	350	128.1
31	65	7.46	378	42.3	30.3	2.31	Not used	5.13	1282	0.200	550	66.8
32	68	7.41	304	43.3	27.1	0.74	0.88	4.90	1225	0.132	300	-

S7. Data from the first study (16 pigs that completed the first study)

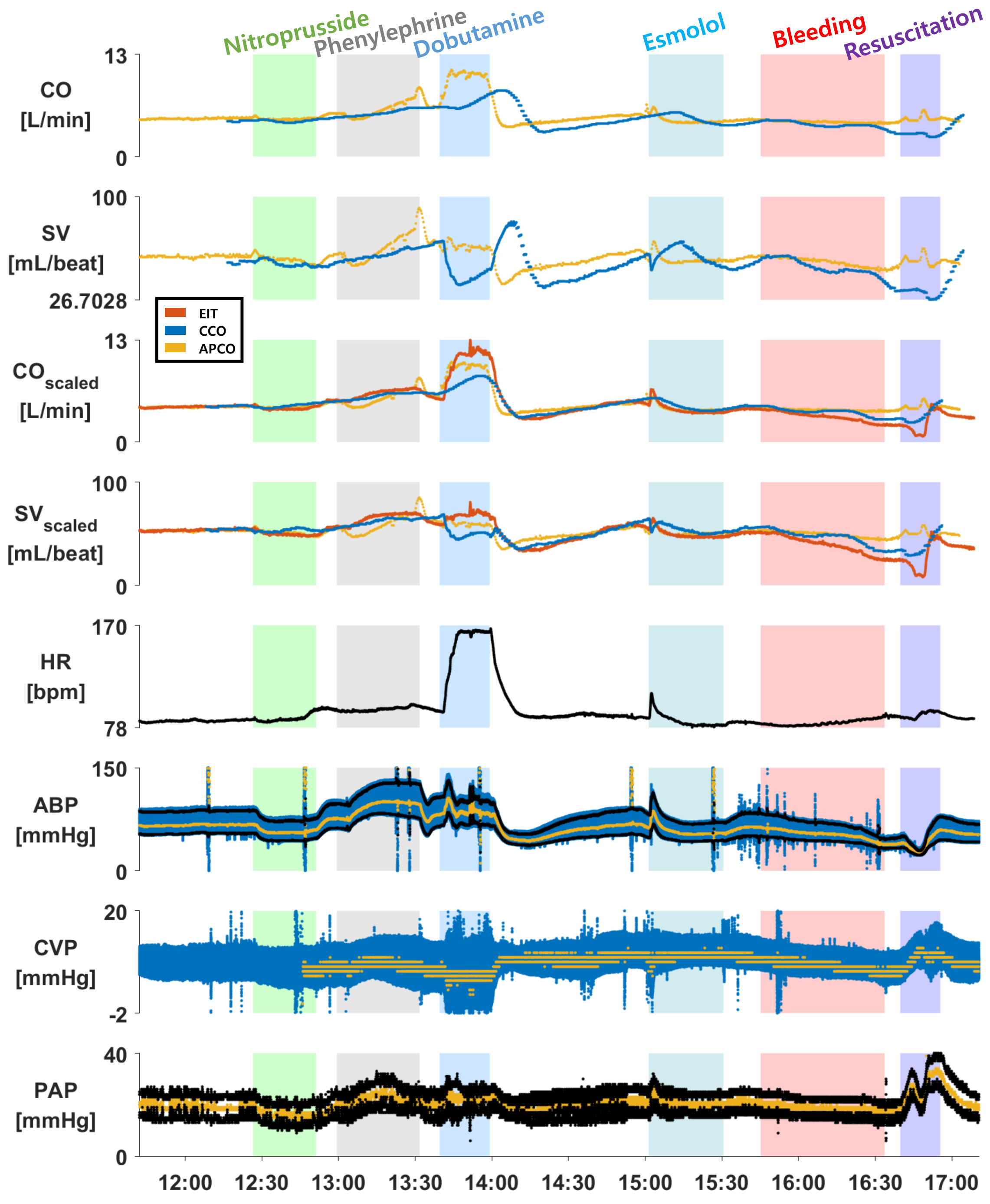

(a) Pig #5 (from 11:42 to 17:10 [hh:mm]).

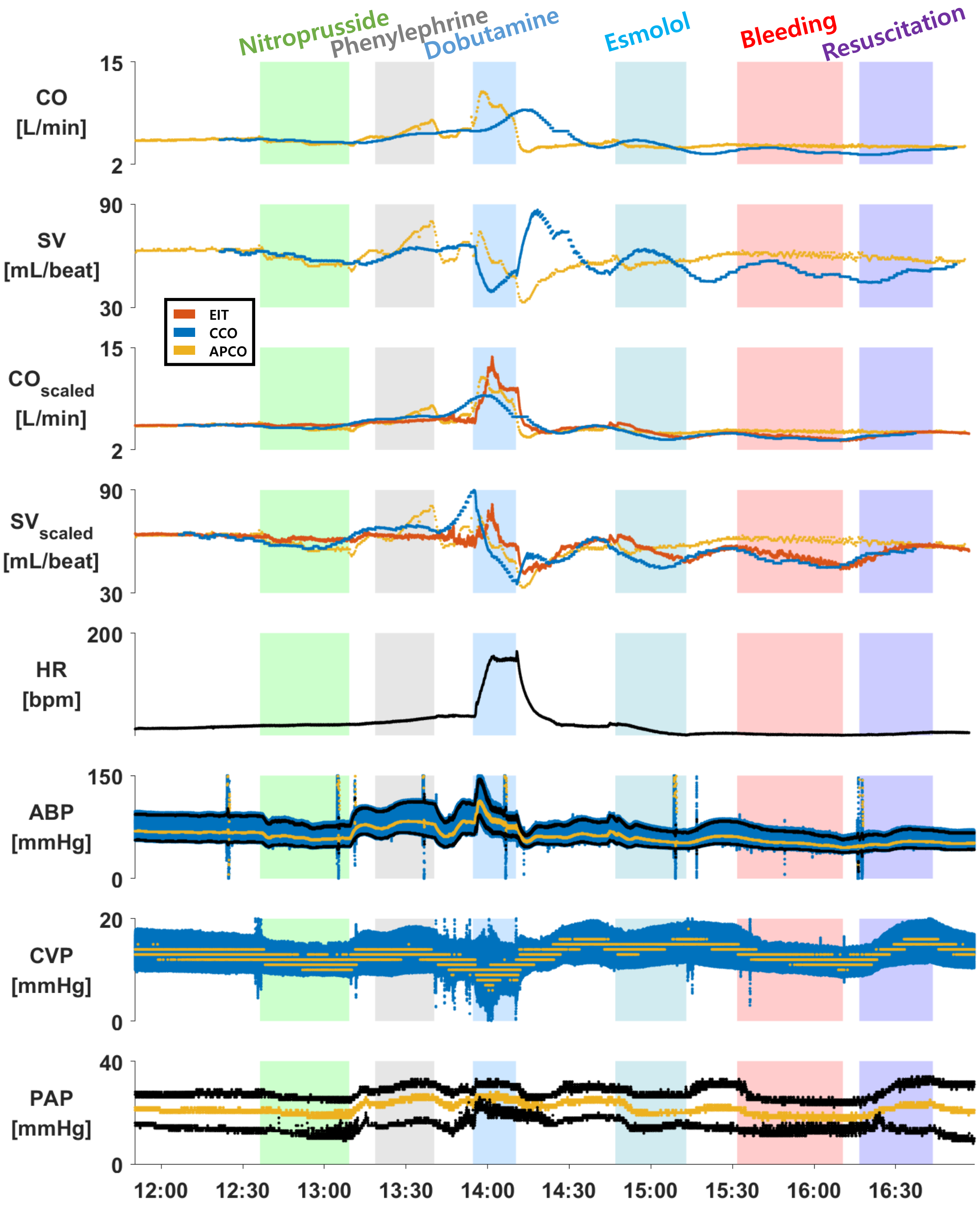

(b) Pig #6 (from 11:50 to 16:58 [hh:mm]).

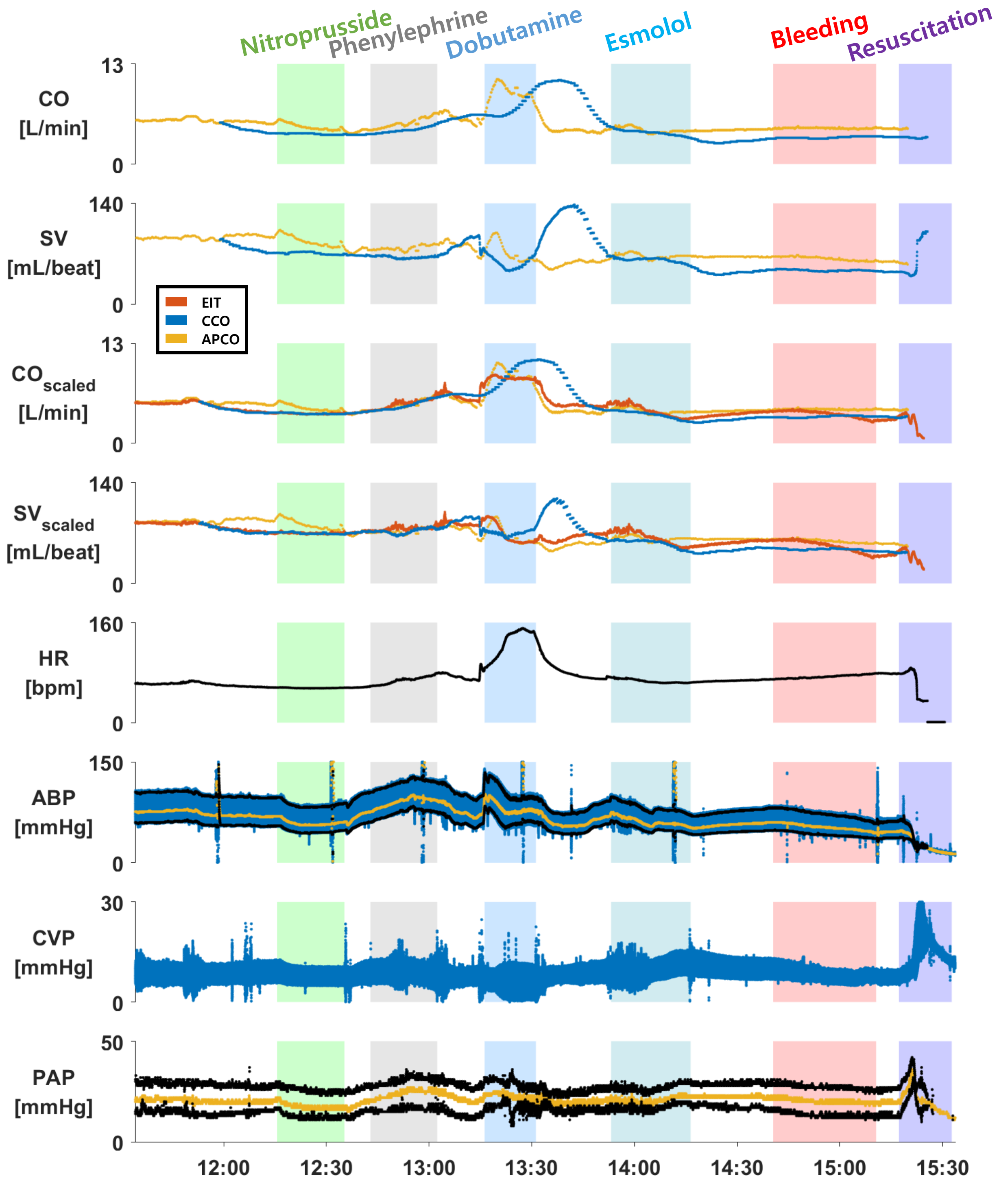

(c) Pig #7 (from 11:34 to 15:33 [hh:mm]).

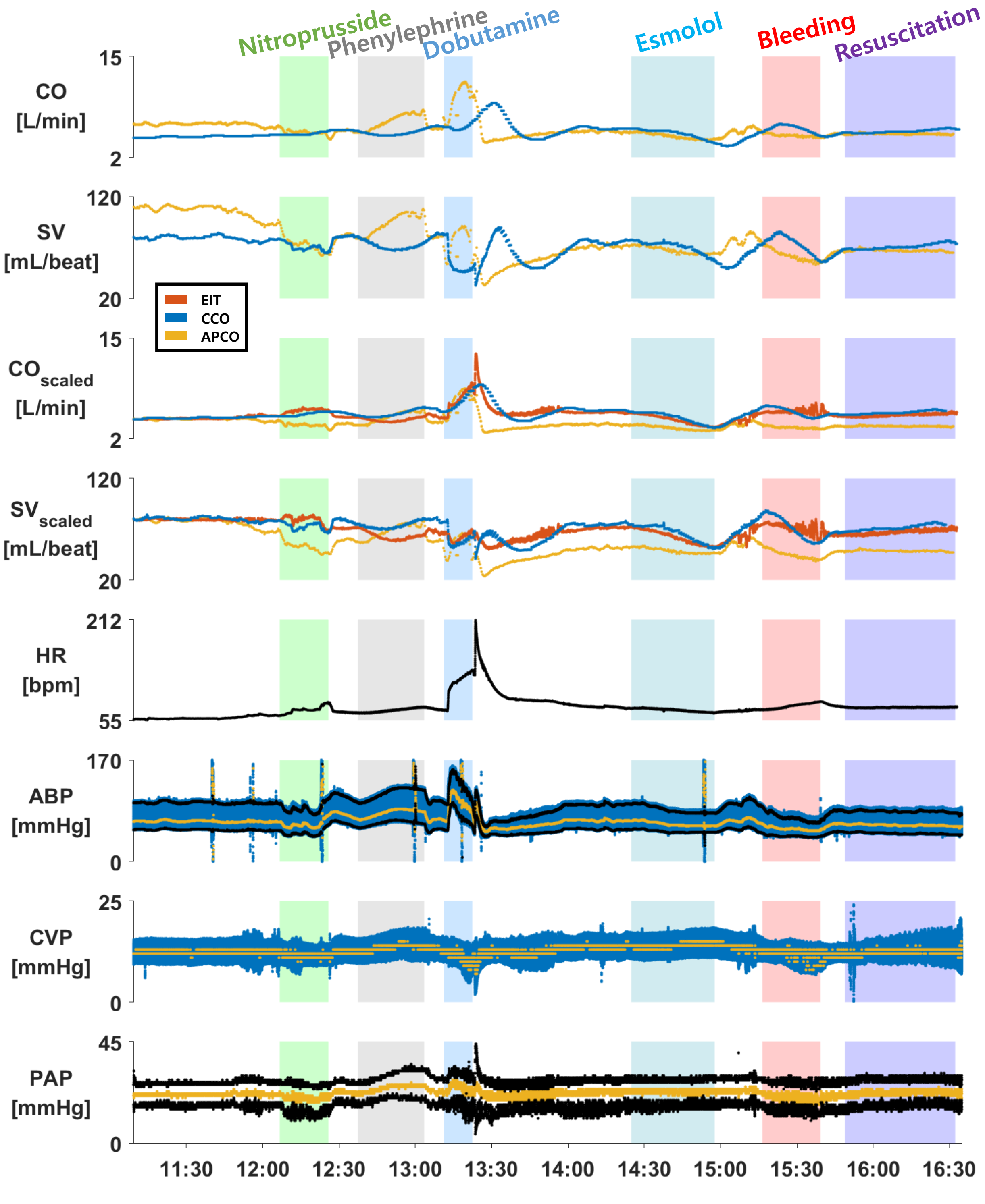

(d) Pig #8 (from 11:09 to 16:34 [hh:mm]).

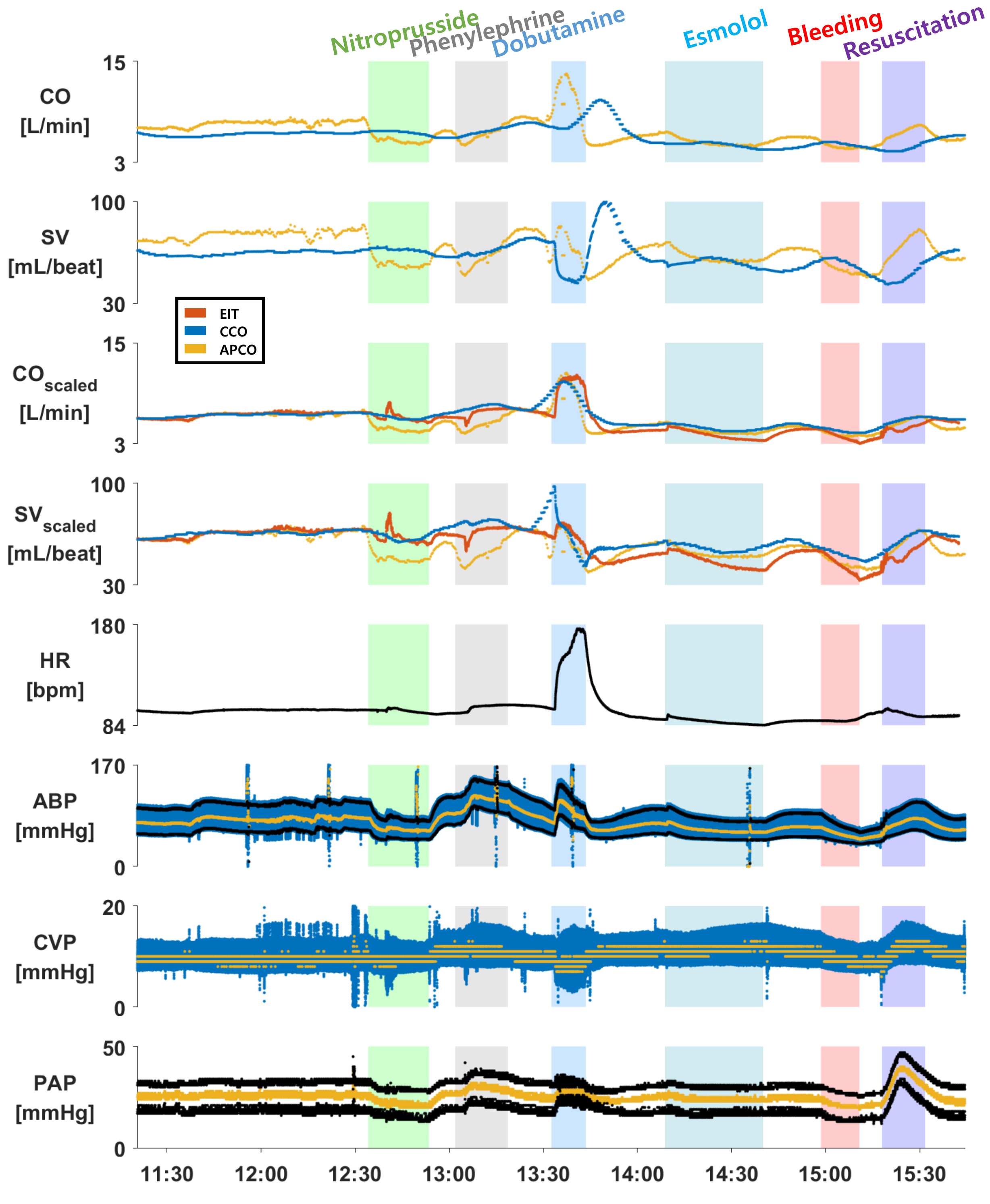

(e) Pig #9 (from 11:20 to 15:44 [hh:mm]).

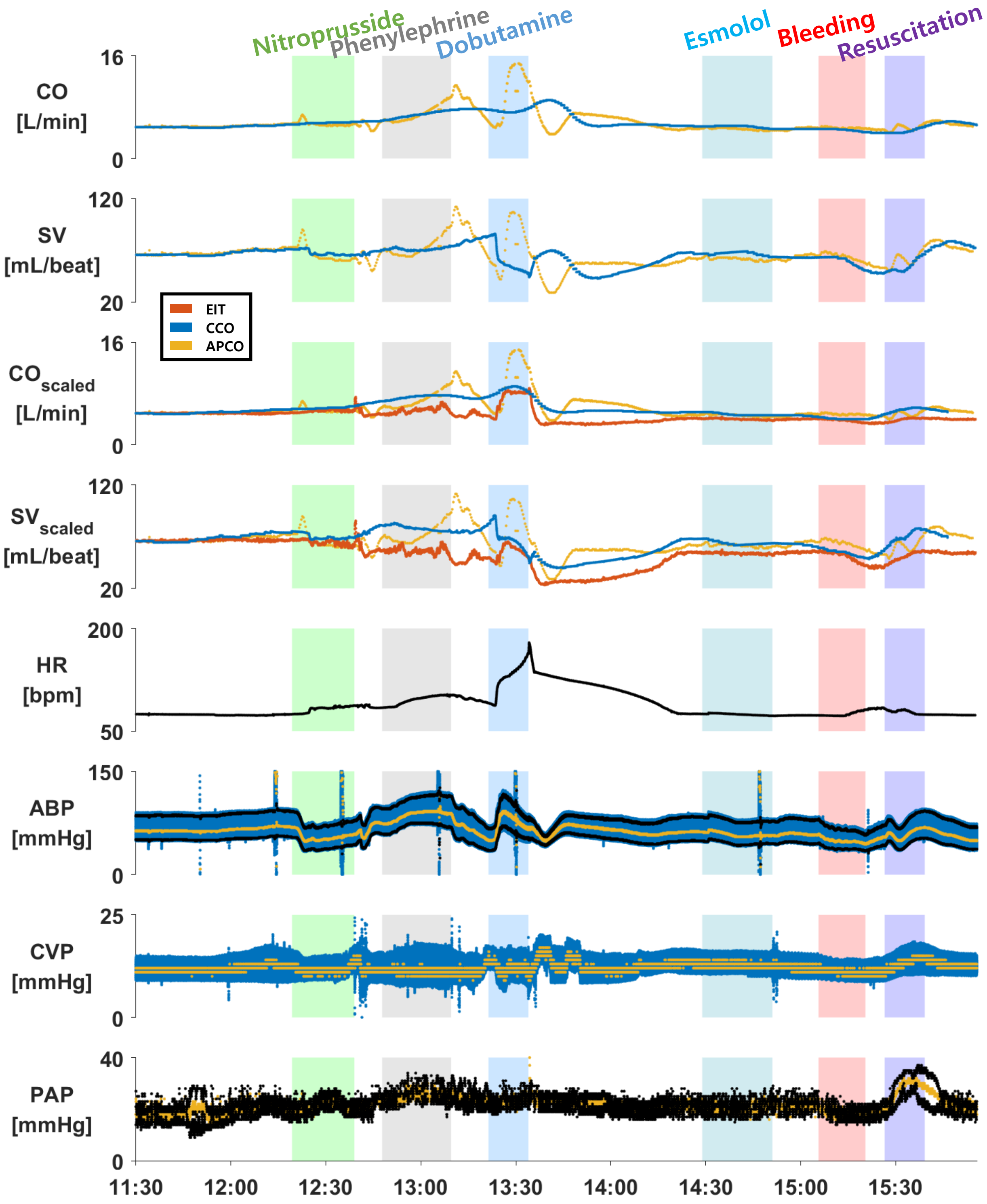

(f) Pig #11 (from 11:30 to 15:55 [hh:mm]).

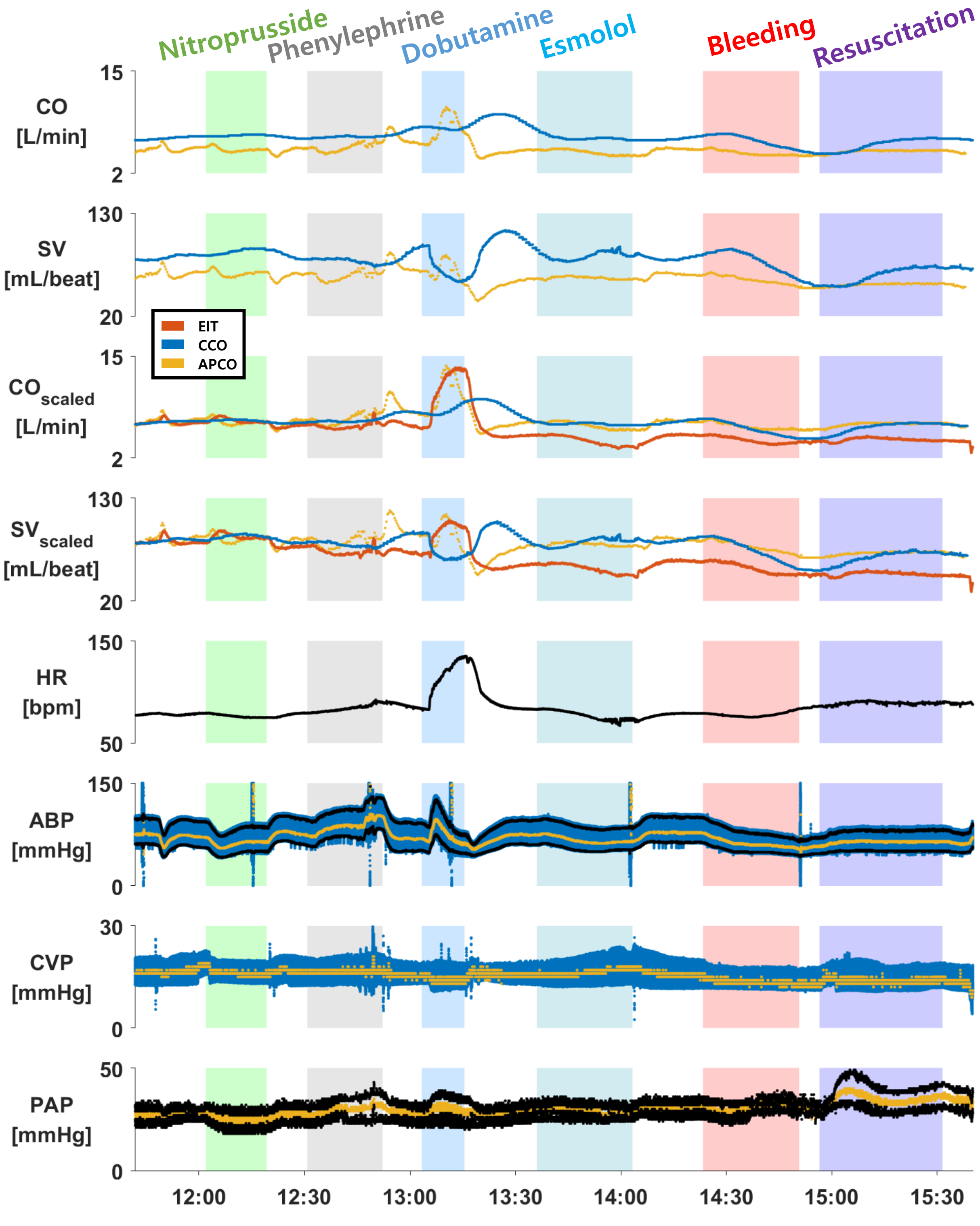

(g) Pig #12 (from 11:41 to 15:40 [hh:mm]).

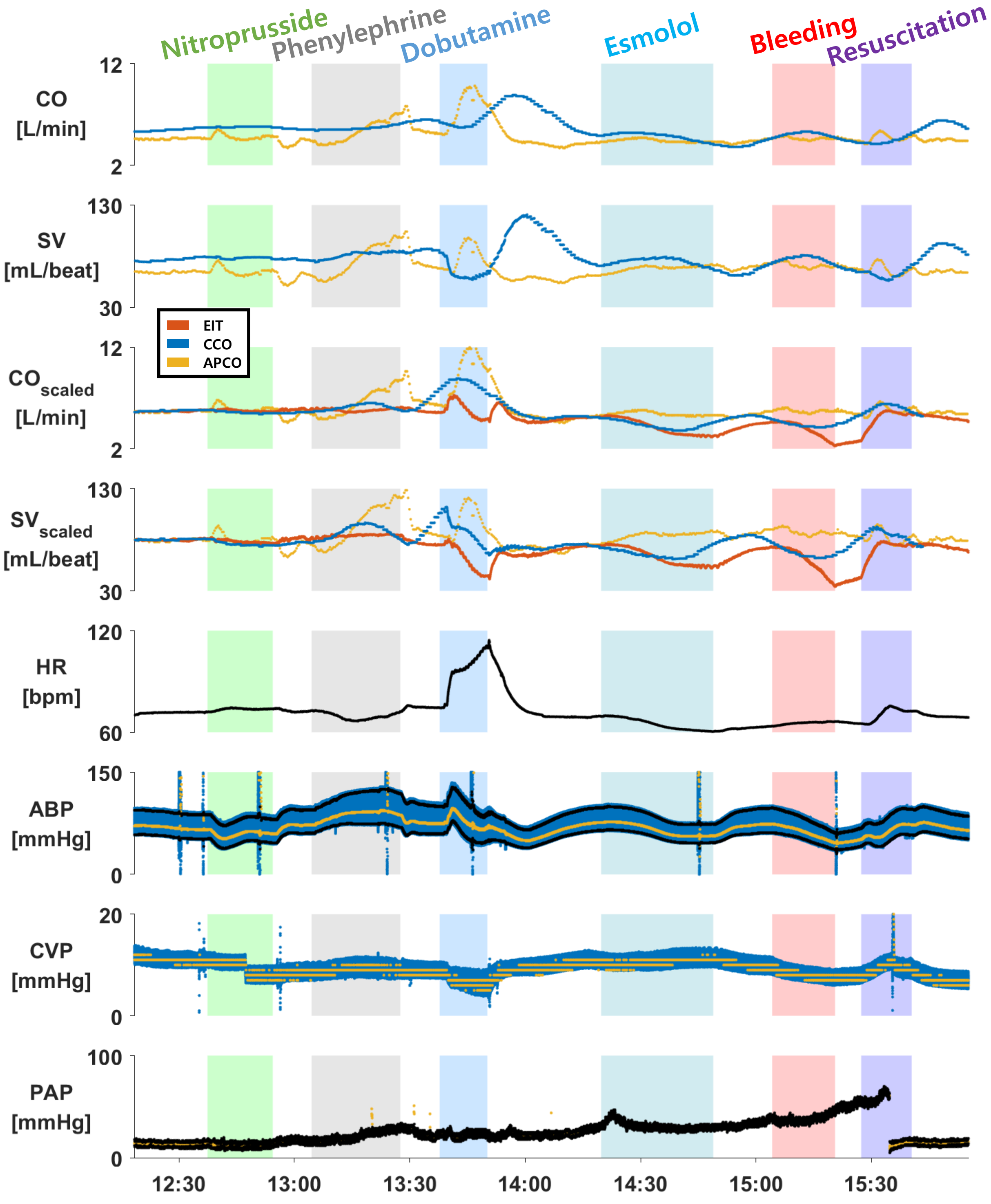

(h) Pig #13 (from 12:18 to 15:55 [hh:mm]). The pulmonary artery catheter was misplaced at 15:32.

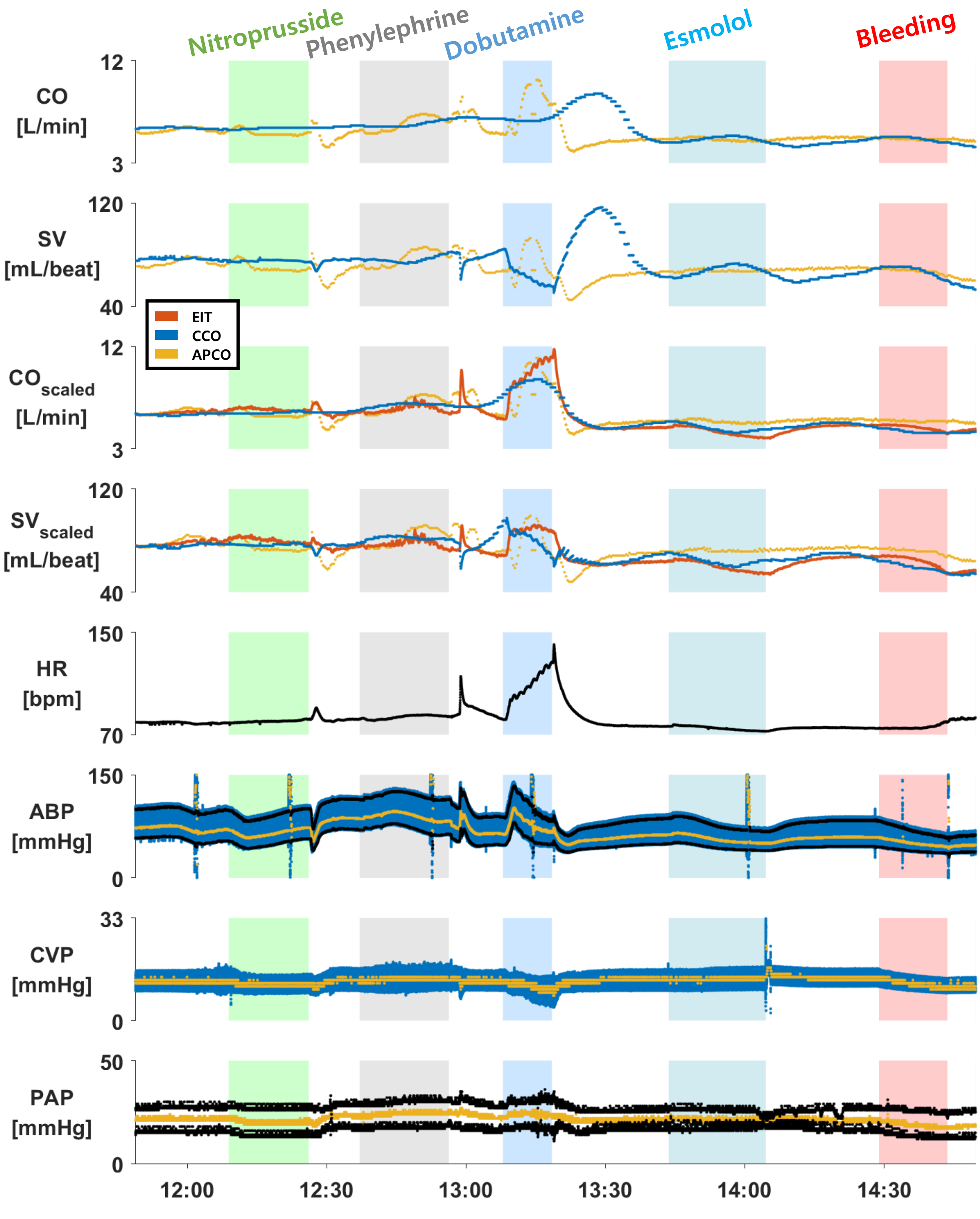

(i) Pig #14 (from 11:48 to 14:49 [hh:mm]).

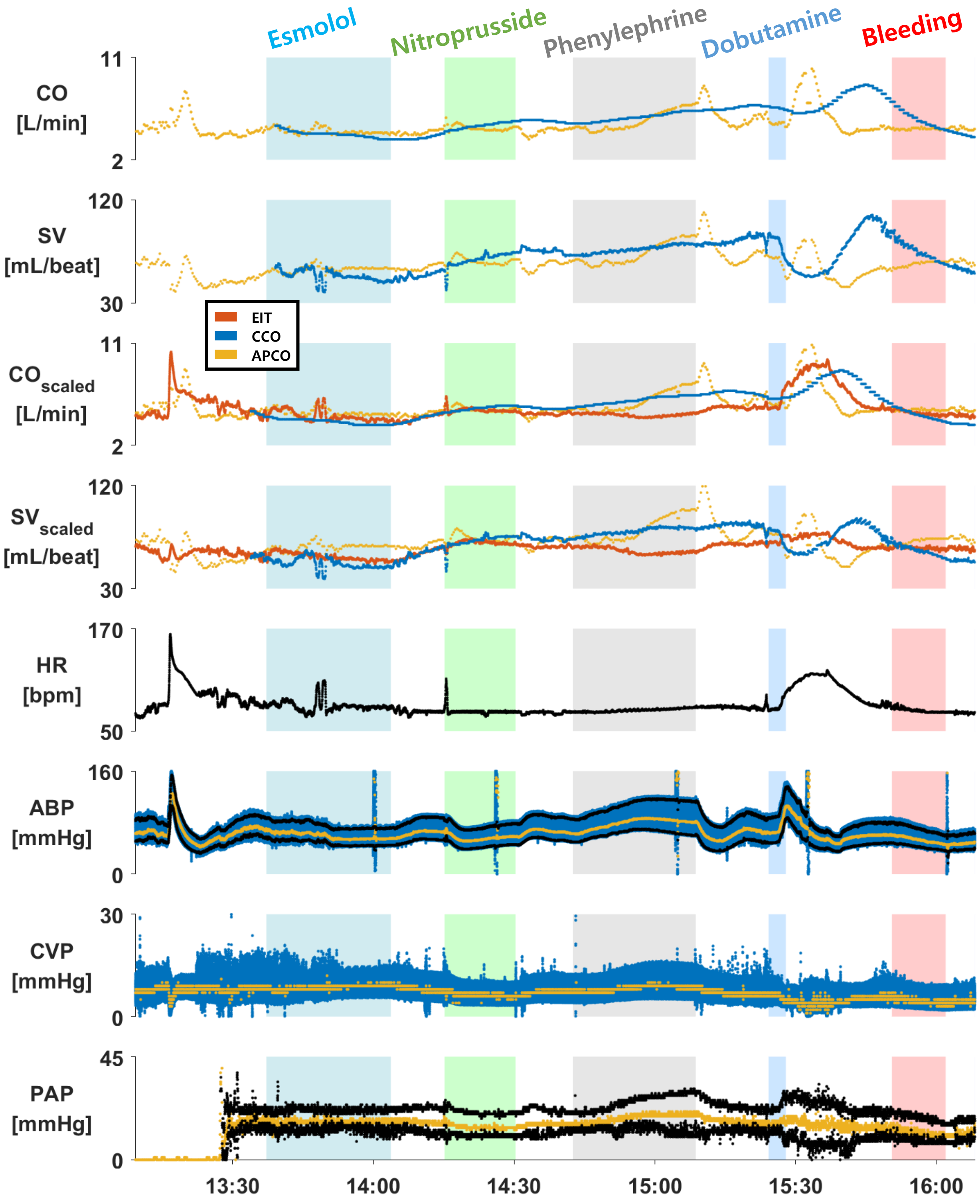

(j) Pig #15 (from 13:09 to 16:08 [hh:mm]). The PAP data were not recorded until 13:28.

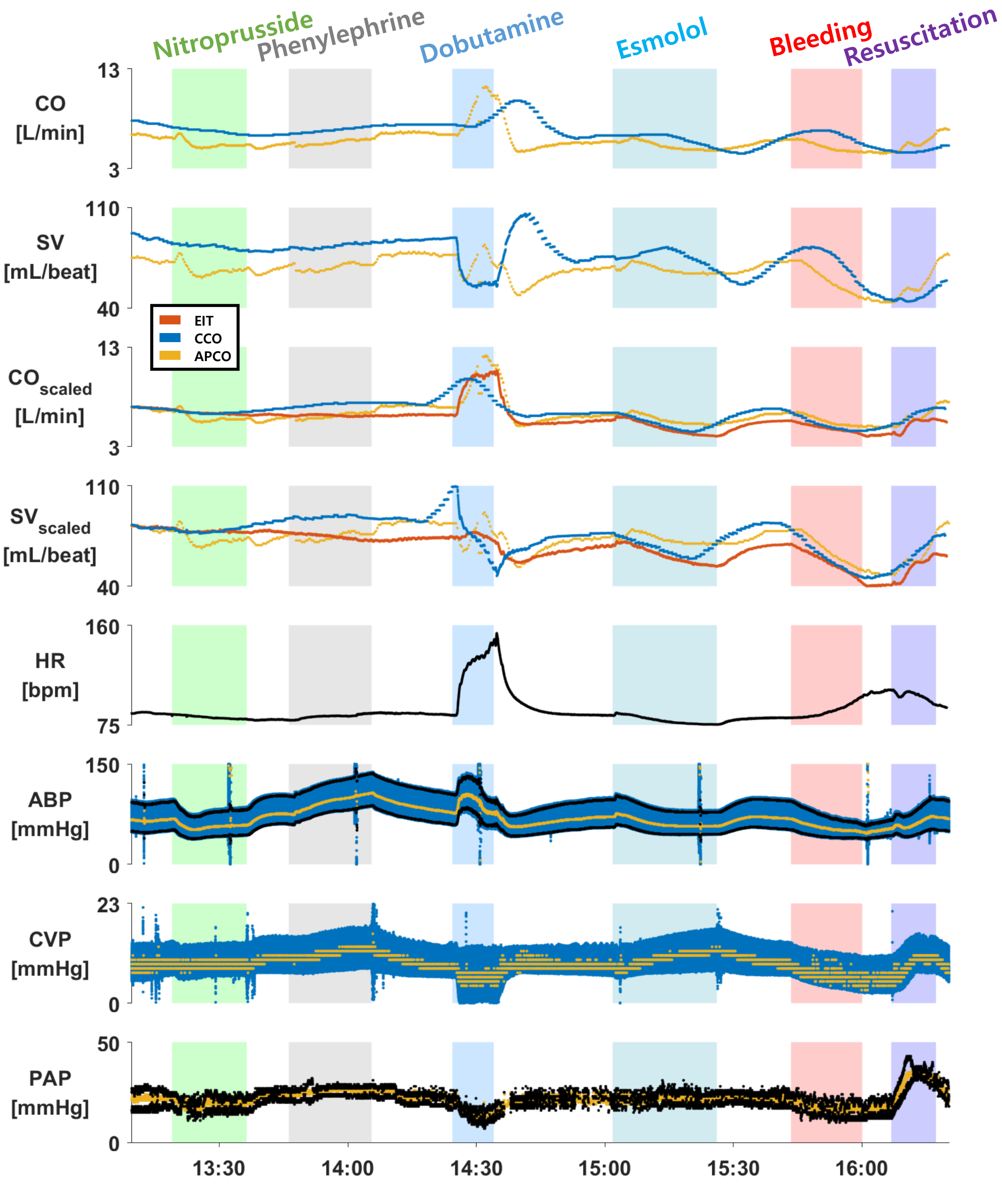

(k) Pig #16 (from 13:09 to 16:20 [hh:mm]).

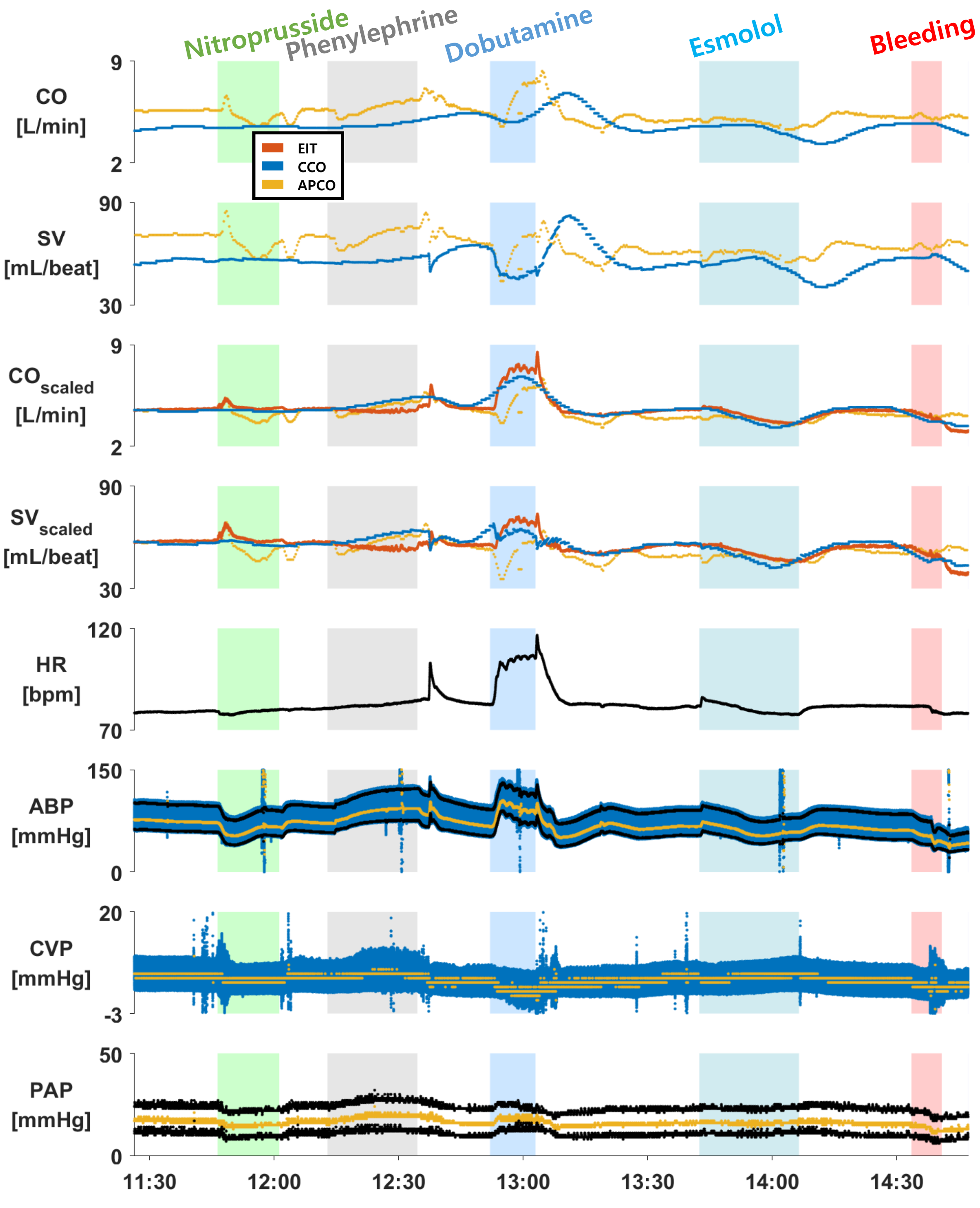

(l) Pig #17 (from 11:26 to 14:47 [hh:mm]).

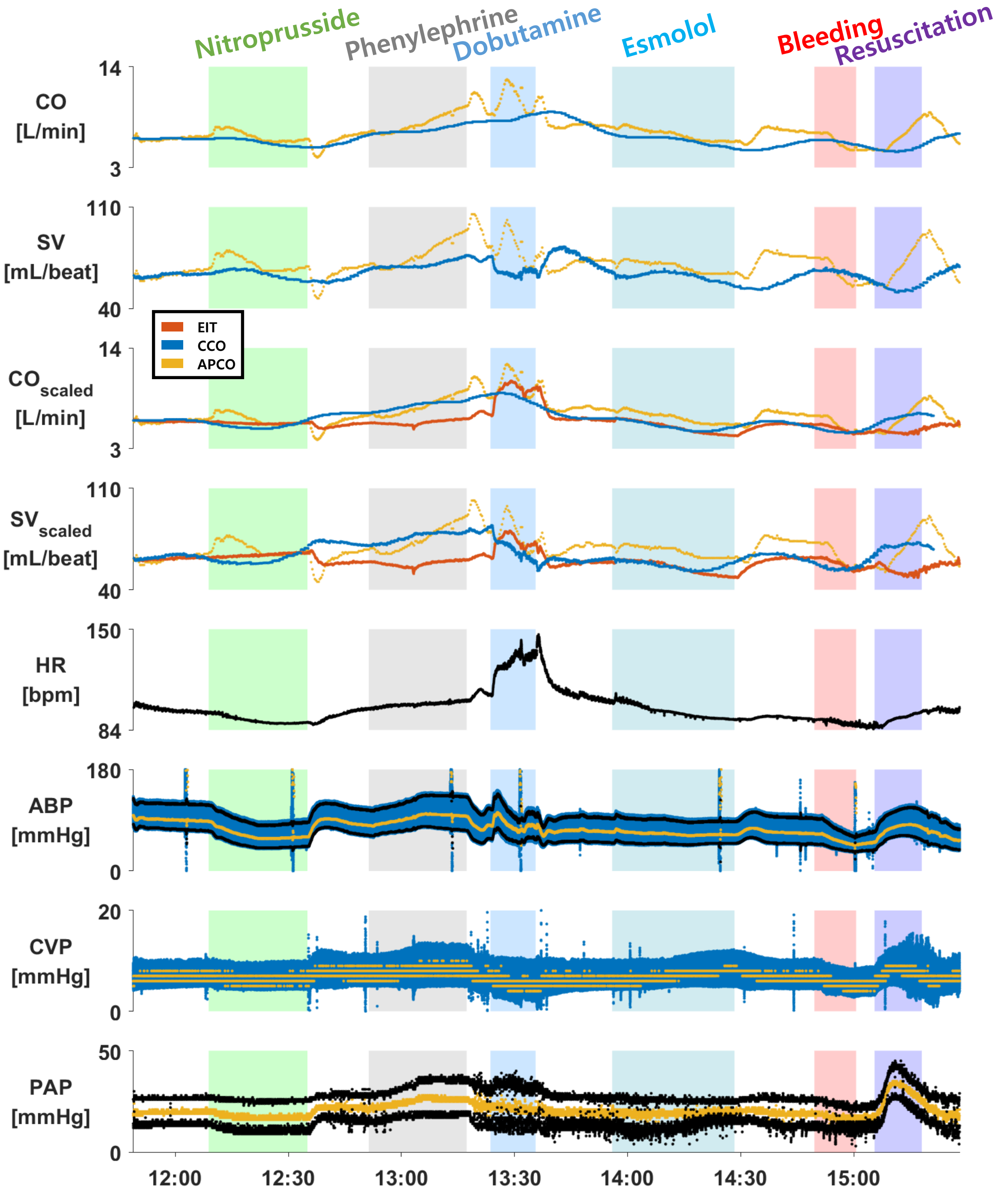

(m) Pig #18 (from 11:48 to 15:28 [hh:mm]).

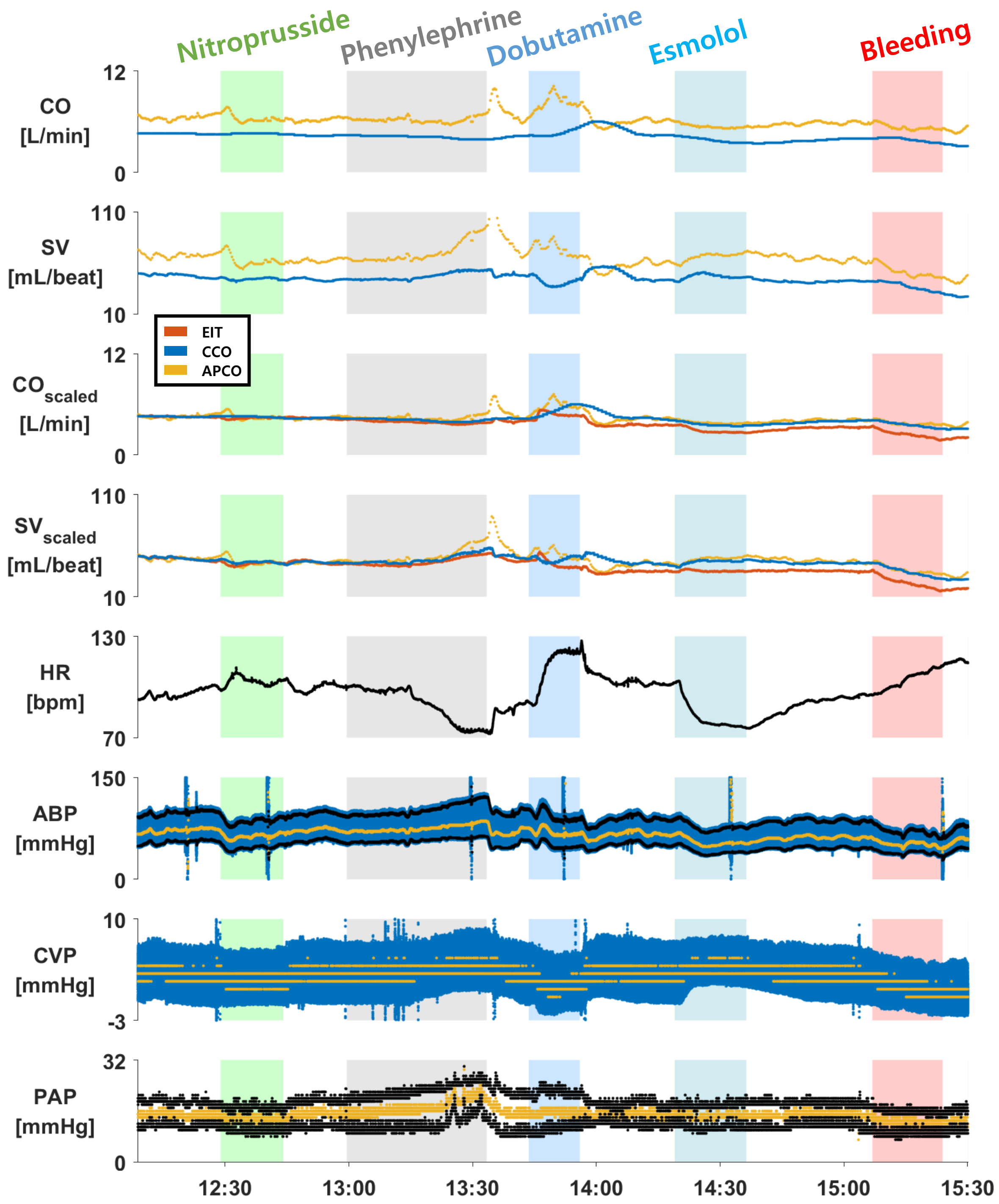

(n) Pig #19 (from 12:08 to 15:30 [hh:mm]).

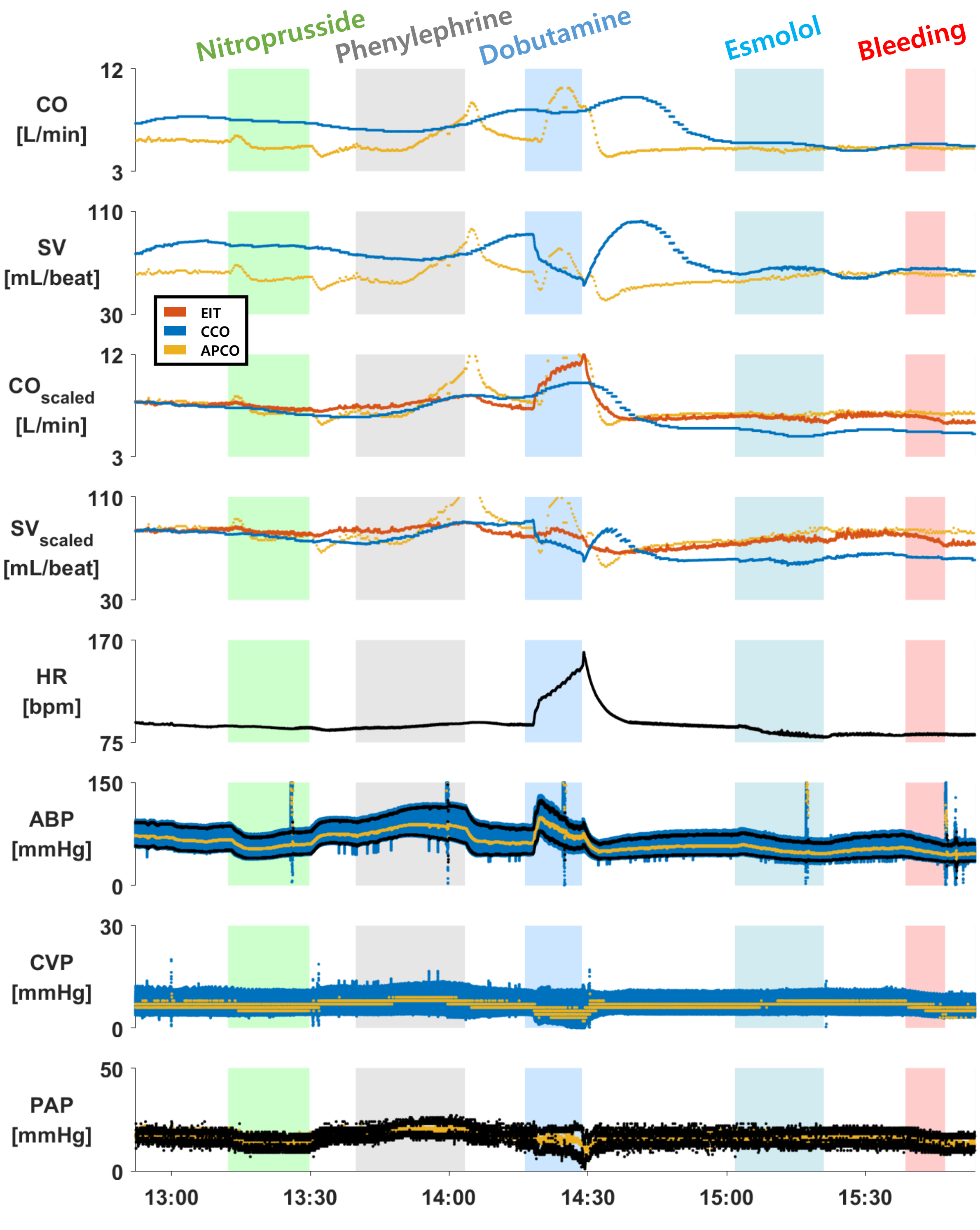

(o) Pig #20 (from 12:52 to 15:53 [hh:mm]).

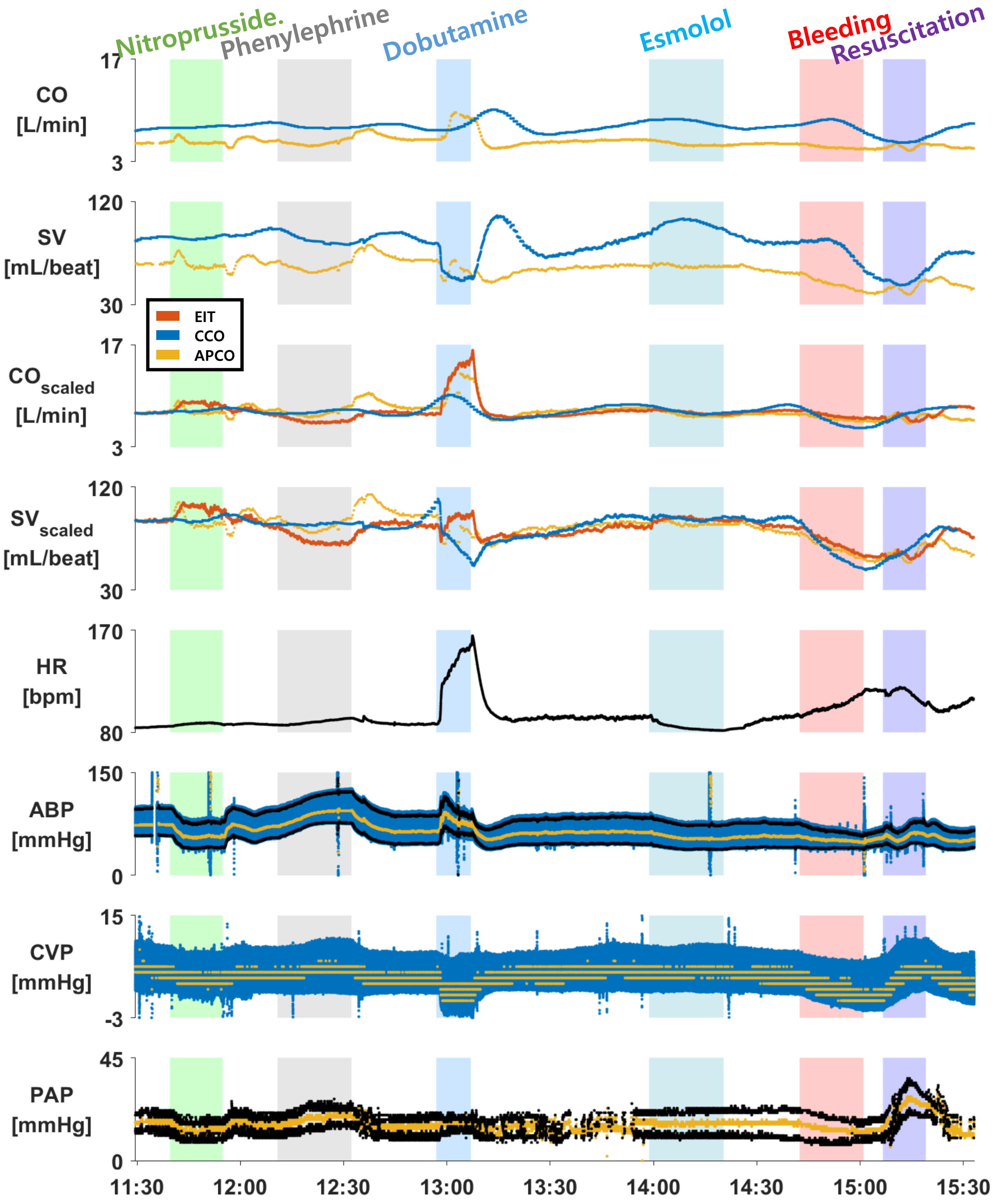

(p) Pig #21 (from 11:29 to 15:33 [hh:mm]).

Fig. S3. (a)~(p) Measured CO, SV, CO_{Scaled}, SV_{Scaled}, HR, ABP, CVP and PAP data for 16 pigs from the first study. In the CO and SV plots, neither amplitude scaling nor time-delay adjustment was applied. In the CO_{Scaled} and SV_{Scaled} plots, the EIT and APCO data were scaled in amplitude using a PAC-CCO datum in the beginning of each experiment as a reference value, and a time-delay adjustment was applied to the PAC-CCO data. The mean values of the ABP, CVP and PAP data are shown in yellow. SV plots were smoothed to remove short-term SV variations.

S8. Data from the second study (7 pigs that completed the second study)

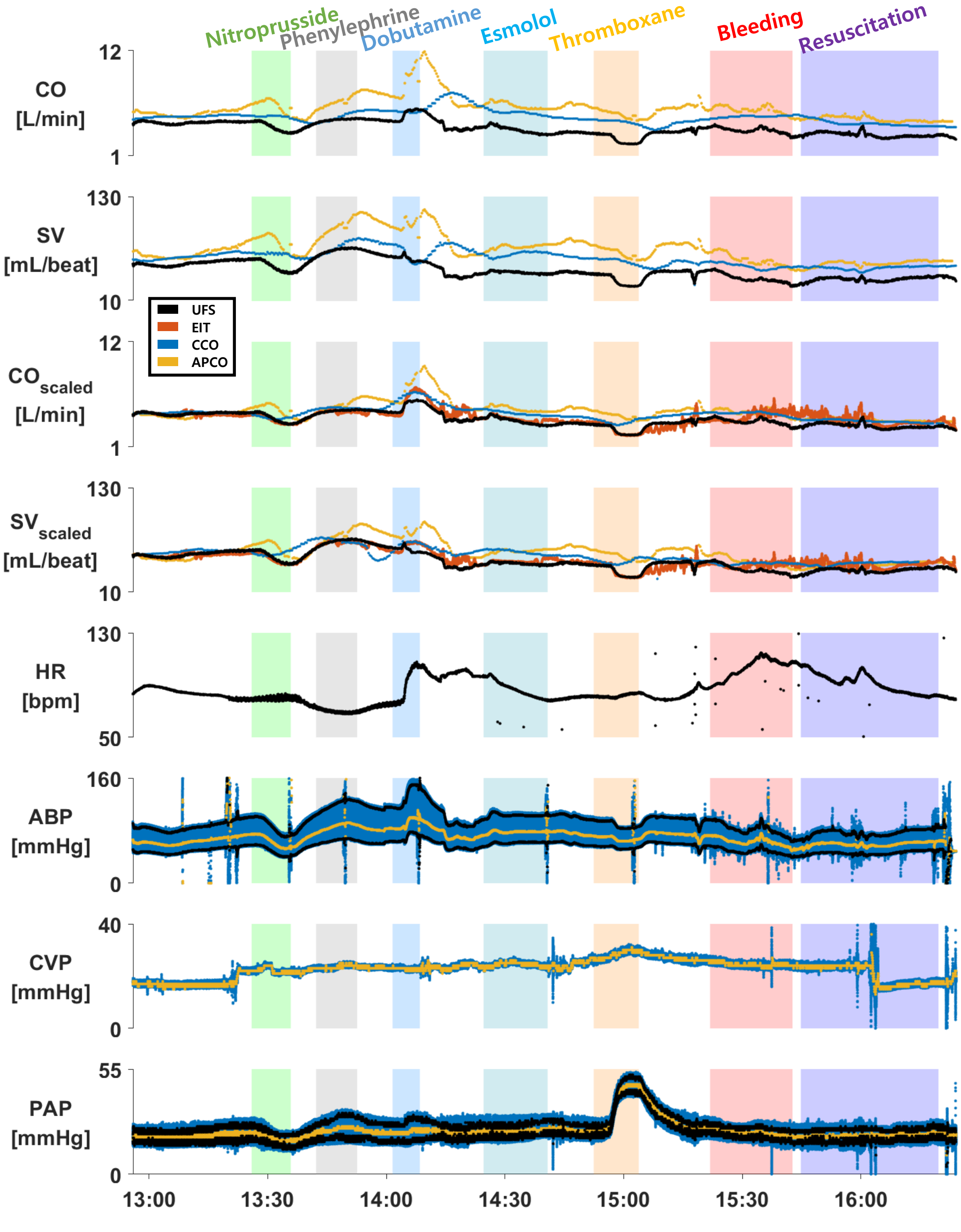

(a) Pig #23 (from 12:55 to 16:24 [hh:mm]).

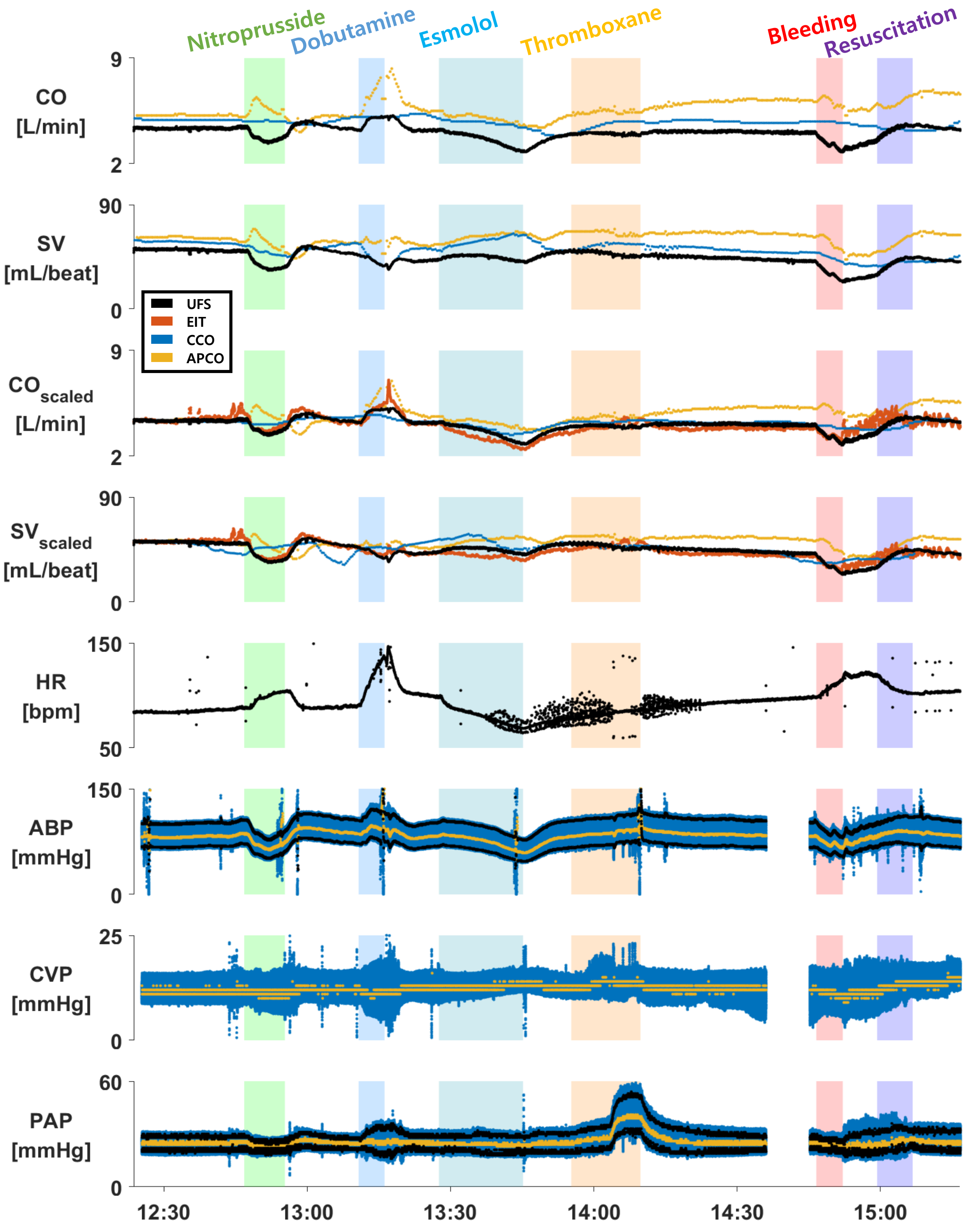

(b) Pig #25 (from 12:23 to 15:16 [hh:mm]). The blood pressure data were not recorded between 14:35 and 14:45.

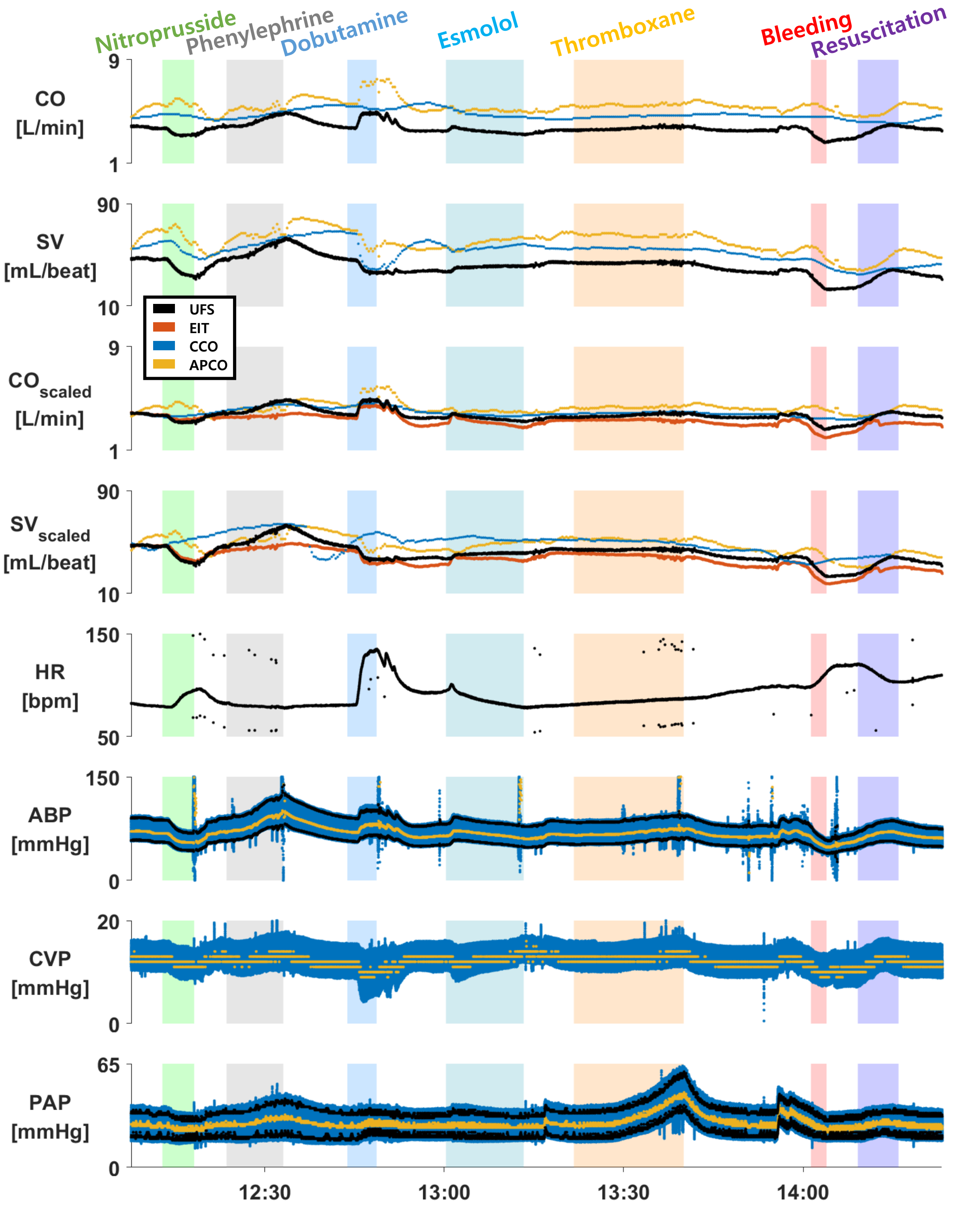

(c) Pig #26 (from 12:07 to 14:23 [hh:mm]).

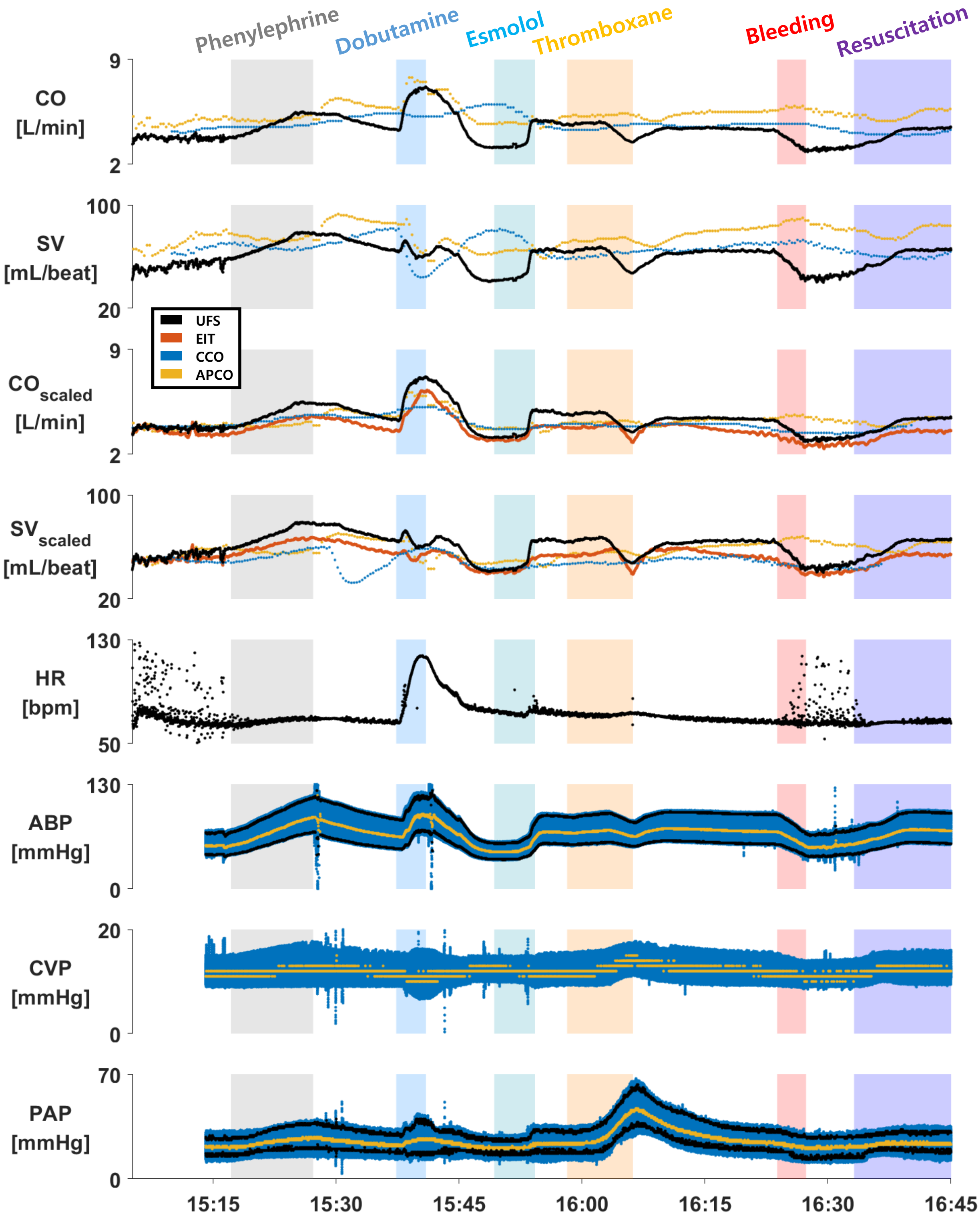

(d) Pig #27 (from 15:05 to 16:45 [hh:mm]). The blood pressure data were not recorded until 15:14.

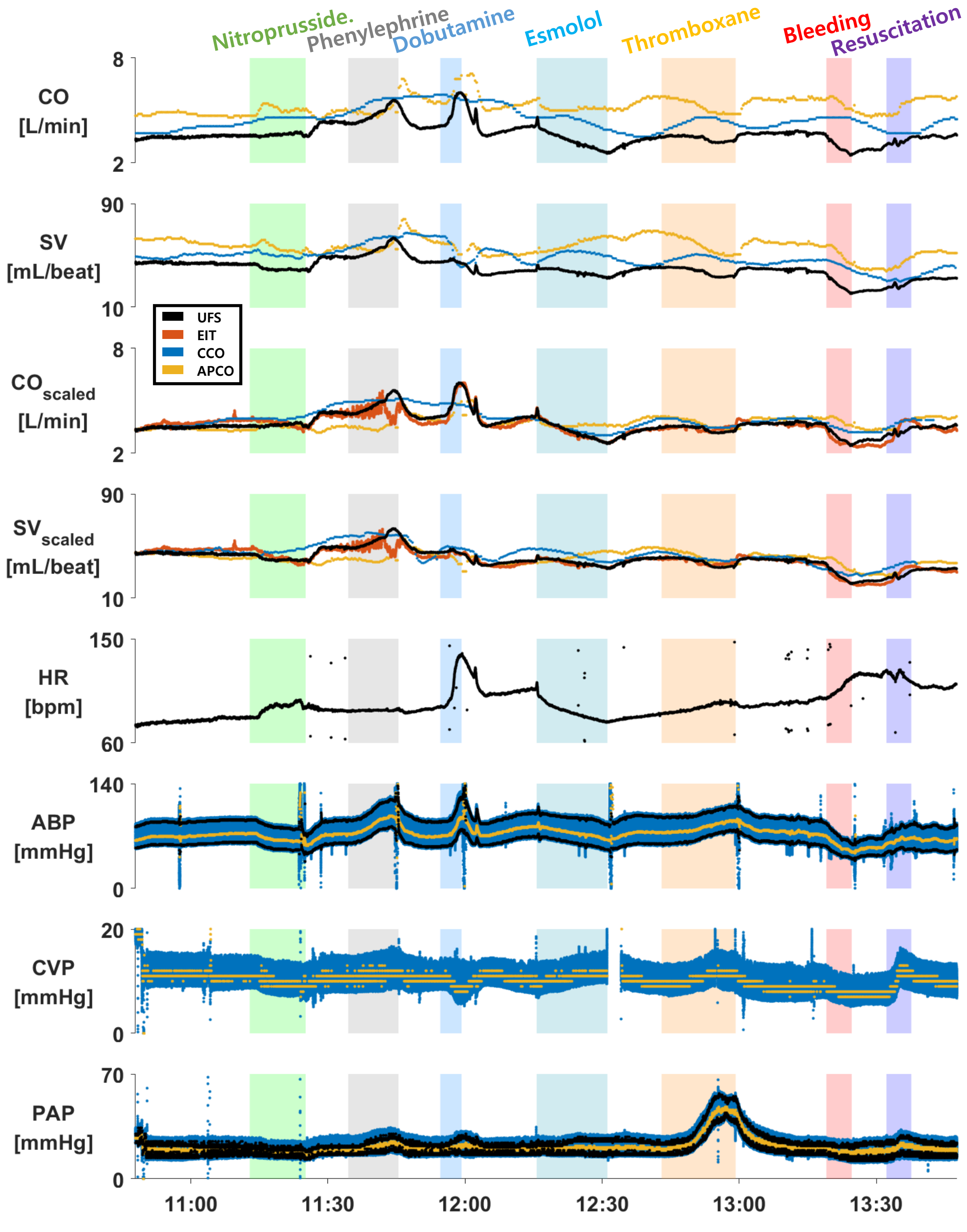

(e) Pig #28 (from 10:47 to 13:47 [hh:mm]). The CVP data were not recorded between 12:31 and 12:34.

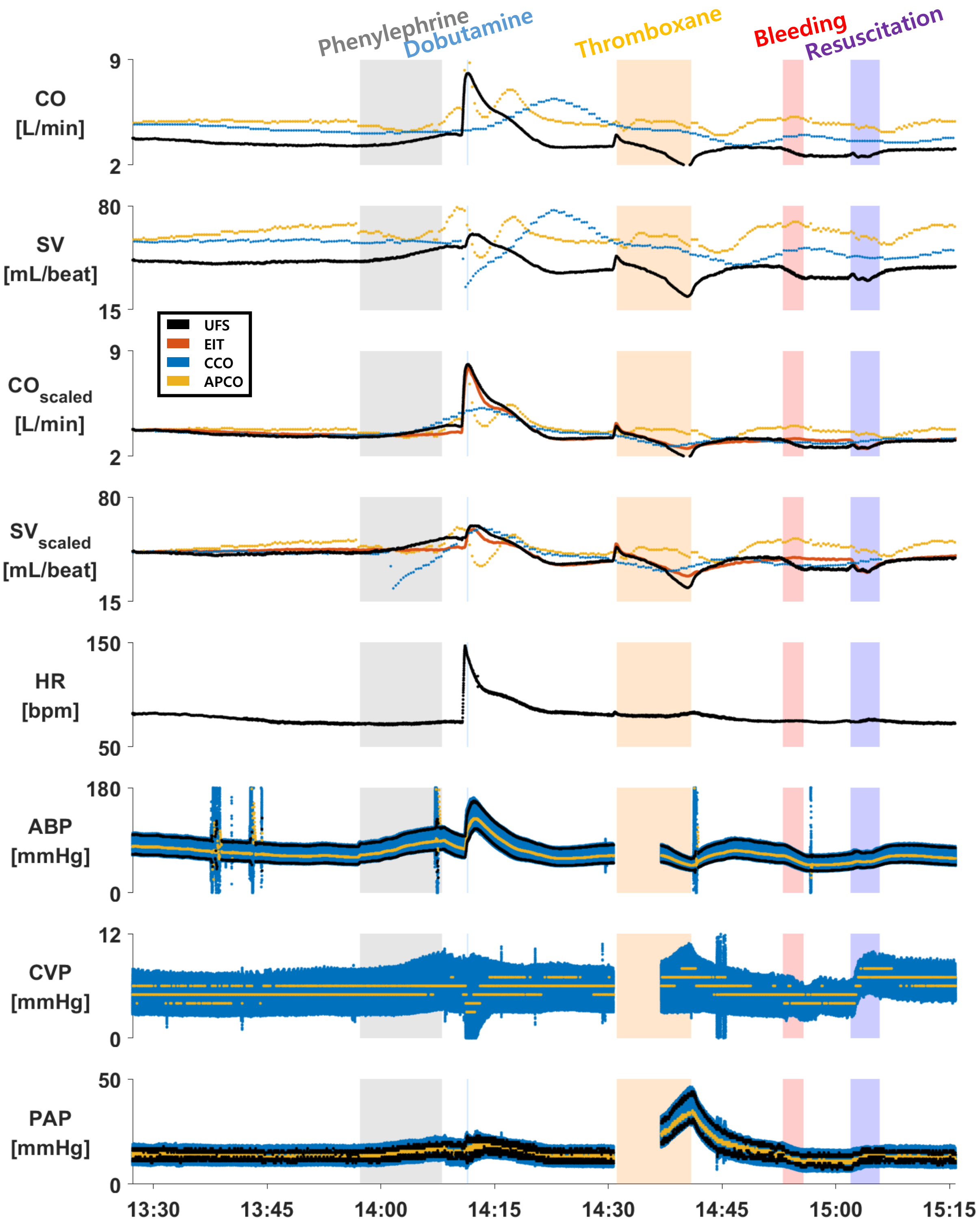

(f) Pig #30 (from 13:27 to 15:16 [hh:mm]). The blood pressure data were not recorded between 14:30 and 14:36.

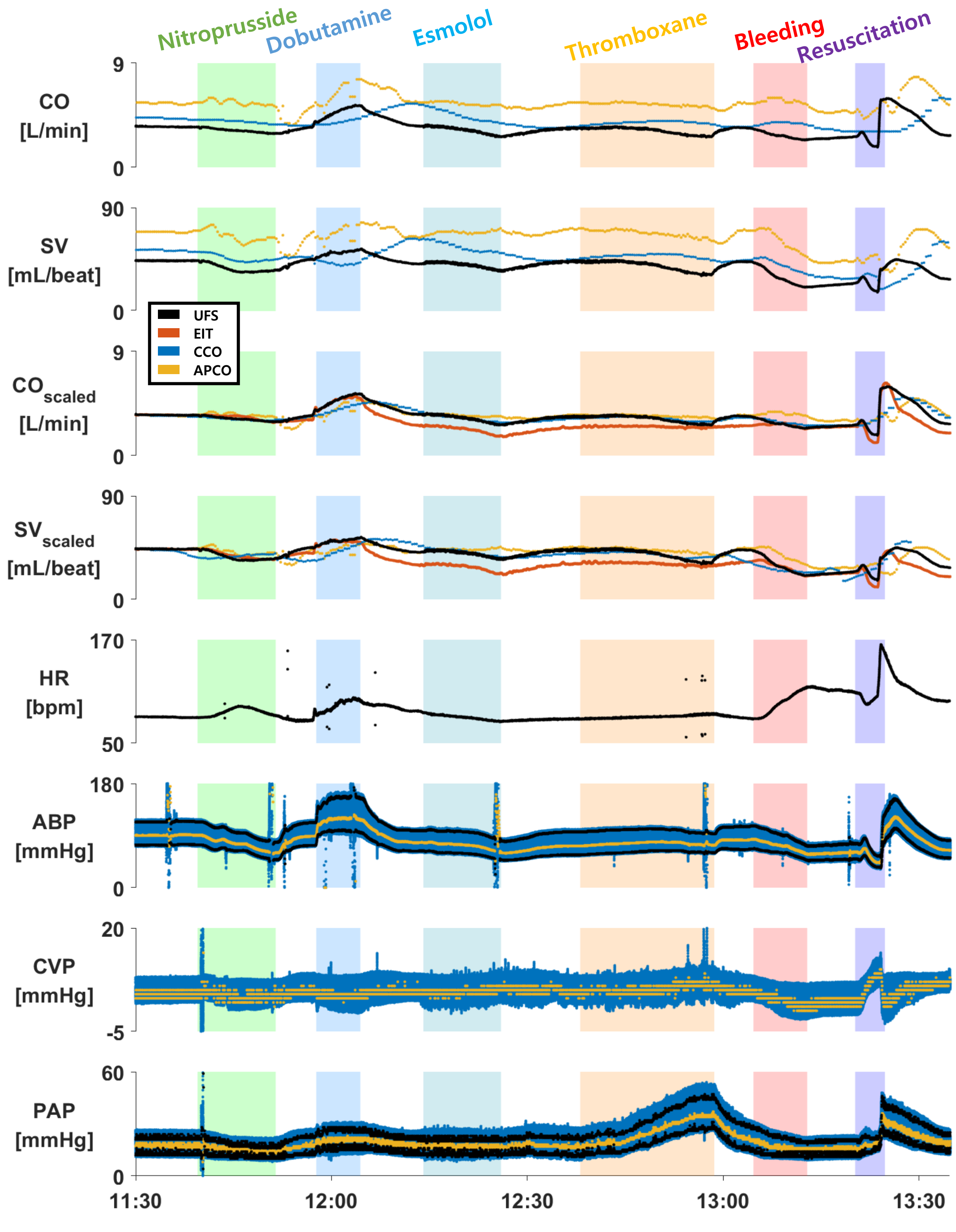

(g) Pig #31 (from 11:30 to 13:34 [hh:mm]).

Fig. S4. (a)~(g) Measured CO, SV, CO_{Scaled}, SV_{Scaled}, HR, ABP, CVP and PAP data for 7 pigs from the second study. In the CO and SV plots, neither amplitude scaling nor time-delay adjustment was applied. In the CO_{Scaled} and SV_{Scaled} plots, the EIT, PAC-CCO and APCO data were scaled using a UFS datum in the beginning of each experiment as a reference value, and a time-delay adjustment was applied to the PAC-CCO data. The mean values of the ABP, CVP and PAP data are shown in yellow. SV plots were smoothed to remove short-term SV variations.

S9. Details about Bland-Altman and Concordance Analyses

Table S2. Details of the Bland-Altman analyses. The results for the APCO method should not be interpreted conclusively due to its unreliability in animals.

X_{REF}	X_{DUT}	Bias, (95% CI)	95% LoA, (95% CI)	PE (%)	# of pigs	# of data pairs	Interval
CO_{UFS}	CO_{EIT}	−0.15, (−0.35, 0.04) L/min	−1.12 and 0.81, (−1.61, 1.30) L/min	26.1	7	12,360	5 sec
CO_{UFS}	$CO_{PAC-CCO}$	0.25, (0.00, 0.49) L/min	−1.23 and 1.72, (−1.75, 2.24) L/min	37.9	7	1,028	1 min
CO_{UFS}	$CO_{PAC-CCO}$ (time delay adjusted)	0.18, (0.02, 0.35) L/min	−0.69 and 1.06, (−1.08, 1.44) L/min	22.7	7	1,006	1 min
CO_{UFS}	CO_{APCO}	0.45, (0.23, 0.67) L/min	−0.87 and 1.77, (−1.36, 2.26) L/min	33.1	7	3,087	20 sec
SV_{UFS}	SV_{EIT}	−1.98, (−4.29, 0.32) mL	−12.8 and 8.80, (−18.6, 14.6) mL	24.8	7	12,360	5 sec
SV_{UFS}	$SV_{PAC-CCO}$	2.89, (0.28, 5.49) mL	−13.1 and 18.9, (−18.8, 24.5) mL	34.8	7	1,028	1 min
SV_{UFS}	$SV_{PAC-CCO}$ (time delay adjusted)	1.75, (−0.85, 4.34) mL	−9.24 and 12.7, (−16.2, 19.6) mL	24.2	7	1,003	1 min
SV_{UFS}	SV_{APCO}	4.49, (1.07, 7.92) mL	−11.6 and 20.6, (−20.2, 29.2) mL	34.5	7	3,087	20 sec
$CO_{PAC-CCO}$	CO_{EIT}	−0.33, (−0.49, −0.17) L/min	−2.66 and 2.00, (−2.86, 2.20) L/min	44.9	23	4,776	1 min
$CO_{PAC-CCO}$	CO_{APCO}	0.05, (−0.12, 0.22) L/min	−2.41 and 2.51, (−2.63, 2.73) L/min	45.8	23	4,781	1 min
$CO_{PAC-CCO}$ (time delay adjusted)	CO_{EIT}	−0.26, (−0.43, −0.10) L/min	−2.06 and 1.54, (−2.30, 1.77) L/min	34.6	23	4,676	1 min
$CO_{PAC-CCO}$ (time delay adjusted)	CO_{APCO}	0.12, (−0.06, 0.30) L/min	−1.76 and 2.00, (−2.01, 2.26) L/min	34.8	23	4,692	1 min
CO_{APCO}	CO_{EIT}	−0.40, (−0.67, −0.13) L/min	−2.67 and 1.87, (−3.10, 2.30) L/min	42.5	23	14,304	20 sec
$SV_{PAC-CCO}$	SV_{EIT}	−4.23, (−6.12, −2.33) mL	−28.9 and 20.5, (−31.5, 23.0) mL	40.6	23	4,785	1 min
$SV_{PAC-CCO}$	SV_{APCO}	0.39, (−1.63, 2.41) mL	−25.4 and 26.2, (−28.2, 28.9) mL	40.9	23	4,773	1 min
$SV_{PAC-CCO}$ (time delay adjusted)	SV_{EIT}	−3.06, (−5.08, −1.05) mL	−22.2 and 16.1, (−25.3, 19.2) mL	31.4	23	4,684	1 min
$SV_{PAC-CCO}$ (time delay adjusted)	SV_{APCO}	1.44, (−0.70, 3.58) mL	−19.4 and 22.3, (−22.6, 25.5) mL	32.9	23	4,684	1 min
SV_{APCO}	SV_{EIT}	−4.38, (−7.39,−1.38) mL	−28.8 and 20.0, (−33.8, 25.0) mL	38.8	23	14,333	20 sec

Table S3. Details of the concordance analyses. The results for the APCO method should not be interpreted conclusively due to its unreliability in animals.

X_{REF}	X_{DUT}	# of pigs	1-minute			5-minute			10-minute		
			**Concord. (%)**	**# of data pairs included**	**# of data pairs**	**Concord. (%)**	**# of data pairs included**	**# of data pairs**	**Concord. (%)**	**# of data pairs included**	**# of data pairs**
CO_{UFS}	CO_{EIT}	7	74.4	90	1,032	88.8	80	207	92.5	53	104
	$CO_{PAC-CCO}$	7	27.8	18	1,026	41.1	56	205	51.8	56	102
	$CO_{PAC-CCO}$ (time delay	7	68.2	22	997	94.5	55	198	92.7	41	102
	CO_{APCO}	7	74.4	43	1,029	62.2	74	205	69.8	53	102
SV_{UFS}	SV_{EIT}	7	80.7	88	1,032	88.7	71	207	94.2	52	104
	$SV_{PAC-CCO}$	7	52.9	34	1,026	61.2	67	205	66.1	56	102
	$SV_{PAC-CCO}$ (time delay	7	65.5	29	997	86.4	59	198	91.3	46	102
	SV_{APCO}	7	55.1	49	1,029	60.8	74	205	71.7	53	102
$CO_{PAC-CCO}$	CO_{EIT}	23	32.0	97	4,759	40.9	186	940	58.3	204	466
	CO_{APCO}	23	32.6	92	4,772	46.0	200	957	54.1	196	484
$CO_{PAC-CCO}$ (time delay adjusted)	CO_{EIT}	23	68.6	102	4,649	78.9	171	915	81.1	164	451
	CO_{APCO}	23	71.0	107	4,673	69.7	185	932	73.3	180	468
CO_{APCO}	CO_{EIT}	23	64.0	247	4,753	70.4	233	938	73.1	201	464
$SV_{PAC-CCO}$	SV_{EIT}	23	45.6	169	4,772	46.1	217	950	50.5	212	476
	SV_{APCO}	23	50.0	142	4,760	55.0	238	946	57.9	214	472
$SV_{PAC-CCO}$ (time delay adjusted)	SV_{EIT}	23	51.0	143	4,662	68.9	183	924	72.4	181	460
	SV_{APCO}	23	55.7	122	4,663	62.4	205	923	66.3	187	458
SV_{APCO}	SV_{EIT}	23	53.2	216	4,767	66.7	231	948	69.3	192	474
